# Metal–Organic Frameworks in Surface Enhanced Raman Spectroscopy–Based Analysis of Volatile Organic Compounds

**DOI:** 10.1002/advs.202401437

**Published:** 2024-06-13

**Authors:** Juan A. Allegretto, Jakub Dostalek

**Affiliations:** ^1^ Laboratory for Life Sciences and Technology (LiST), Department of Medicine, Faculty of Medicine and Dentistry Danube Private University Krems 3500 Austria; ^2^ FZU‐Institute of Physics Czech Academy of Sciences Na Slovance 2 Prague 82021 Czech Republic

**Keywords:** MOF, Raman, sensing, SERS, volatile organic compounds

## Abstract

Volatile Organic Compounds (VOC) are a major class of environmental pollutants hazardous to human health, but also highly relevant in other fields including early disease diagnostics and organoleptic perception of aliments. Therefore, accurate analysis of VOC is essential, and a need for new analytical methods is witnessed for rapid on‐site detection without complex sample preparation. Surface‐Enhanced Raman Spectroscopy (SERS) offers a rapidly developing versatile analytical platform for the portable detection of chemical species. Nonetheless, the need for efficient docking of target analytes at the metallic surface significantly narrows the applicability of SERS. This limitation can be circumvented by interfacing the sensor surface with Metal–Organic Frameworks (MOF). These materials featuring chemical and structural versatility can efficiently pre‐concentrate low molecular weight species such as VOC through their ordered porous structure. This review presents recent trends in the development of MOF‐based SERS substrates with a focus on elucidating respective design rules for maximizing analytical performance. An overview of the status of the detection of harmful VOC is discussed in the context of industrial and environmental monitoring. In addition, a survey of the analysis of VOC biomarkers for medical diagnosis and emerging applications in aroma and flavor profiling is included.

## Graphical abstract

The integration of Metal–Organic Frameworks (MOF) into Surface‐Enhanced Raman Spectroscopy (SERS) sensors is discussed, focusing on the complex interplay of both worlds, and elucidating respective design rules for maximizing the analytical performance of the platform. Application in industrial and environmental monitoring, early disease diagnostics, and aroma and flavor profiling is thoroughly summarized, discussing the relevant design parameters for each case.

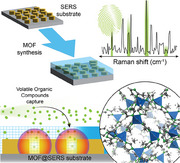

## 1 Introduction

Volatile Organic Compounds (VOC) encompass a diverse group of carbon‐based chemicals that can be found as gases or vapors at ambient temperatures. Although they can originate from natural processes, human activity is assumed to be the major source of VOC that increasingly penetrate our immediate surroundings.^[^ ^1^ ^]^ The raising occurrence of VOC in the environment imposes significant consequences across multiple industrial activities related to the production and/or use of chemicals.^[^ ^2^, ^3^ ^]^ In addition, VOC are relevant to odor pollution associated with composting and solid waste management in landfills.^[^ ^4^, ^5^ ^]^ The analysis of VOC also plays an important role in biomedical applications with a strong focus on the analysis of biomarkers that can be non‐invasively collected from breath for disease diagnosis.^[^ ^6^, ^7^, ^8^ ^]^ Moreover, VOC are key players in the food industry through their inevitable role in organoleptic characteristics (aroma and flavor) and the overall sensory perception of alimentary products.^[^ ^9^, ^10^ ^]^ For example, it has been demonstrated that VOC can persist in plastic containers used for food storage, posing complications to their recycling by potentially altering the perception of the contained food quality.^[^ ^11^ ^]^

Through the past decades, numerous technologies were developed for the analysis of VOC^[^ ^12^ ^]^ and Gas Chromatography (GC) evolved to be the key benchmark method.^[^ ^13^, ^14^ ^]^ GC is based on the vaporization of the sample and its interaction with a stationary phase for separating the sample components before their subsequent detection. Among these, there can be distinguished several strategies in GC analytical technologies with their intrinsic advantages and limitations. To name a few examples, a Flame Ionization Detector provides means of detection with high sensitivity and over wide concentration ranges, but it leads to destruction of the sample and needs using of flammable gases.^[^ ^15^ ^]^ Ion Mobility spectrometry does not require preconcentration of the sample and provides high ppm sensitivity, however, it cannot identify the chemical components and is not suitable for real‐time measurements.^[^ ^16^ ^]^ Mass Spectrometry, perhaps the gold standard for GC‐based detection of analytes, allows for chemical identification and profiling of analytes with excellent sensitivity (ppb range) and reproducibility. However, it relies on complex instrumentation that needs to be operated by highly qualified personnel and employs time‐consuming pretreatment protocols. Altogether, despite the advantages of GC in combination with different detection techniques, the application of GC‐based technology for VOC detection is restricted to dedicated laboratories, where the analyzed sample is transported from the tested spot. Despite the advances in miniaturization and the development of portable MS‐GC, its application for VOC sensing remains elusive.^[^ ^17^ ^]^

Different alternatives have been pursued for the development of simpler and yet robust detection platforms. This includes classical photoionization detectors, where the VOC are ionized, and the generated current is employed as a readout. This approach provides a quantitative information, but typically chemical identification of the analyzed compounds is not possible.^[^ ^18^ ^]^ Electrochemical sensors can also provide quantitative analytical information and can be employed to generate sensor arrays, capable of identifying complex mixtures of VOC as in the case of aromas profiling. For this, specific analyte‐substrate interactions are needed, since it is upon that particular interaction that electrochemical signal is generated and later converted into the sensor´s readout. This requires the sensor to be highly selective thus avoiding unspecific interactions that mask the analyte signal, especially when handling trace concentrations.^[^ ^19^ ^]^ As a way to circumvent these limitations, spectroscopic techniques can be employed, and among these Surface‐Enhanced Raman Spectroscopy (SERS) has been proven to hold a prominent stage in highly sensitive and specific detection of target species.^[^ ^20^ ^]^ Here the fingerprinting capability of SERS is employed as optical readout, univocally identifying the analyte. Hence analyte‐substrate optimization is required now for maximizing the performance of the substrate, as will be discussed later. SERS represents a rapidly developing technology area that capitalizes on the establishing of well‐controlled routes for the preparation of metallic nanostructures with tailored optical properties. SERS is a method providing the means for direct fingerprinting of target molecules with fast detection times, possibility of in situ and real‐time sampling, and facile integration with other complementary detection techniques.^[^ ^21^, ^22^, ^23^ ^]^ The drawback of considering SERS for VOC detection is the need of the attachment of the target analyte to close proximity to SERS‐active surface carrying metallic nanostructures. Thus, if the analyte does not chemically react with the metallic surface such as gold or silver, it cannot be directly detected, and additional strategies need to be employed for its trapping. One approach is to condense the vapor or gas sample to liquid phase that is subsequently contacted with the sensor surface.^[^ ^24^, ^25^ ^]^ However, if target species are present at low concentrations, such preconcentration is not sufficient and more efficient means for selective entrapment of analytes at the SERS‐active surface is needed.

A promising solution lies in the combination of metallic structures with Metal‐Organic Frameworks (MOF). These materials emerged by the end of the ′90s and utilize an elegant principle of coordination of metallic centers by a bi‐ or multidentate ligand.^[^ ^26^, ^27^ ^]^ Each of the coordination points interacts with a different metallic unit instead of chelating the initial metal, which then propagates a network of coordinative bonds, creating extended structures.^[^ ^28^ ^]^ Among the different main classes of MOF, those crystalline and featuring permanent porosity are of particular interest given their extraordinarily large surface areas, that can reach values up to almost 8000 m^2^g^−1^.^[^ ^29^ ^]^ Despite their complexity, they can be synthesized by flexible routes in a wide range of conditions (in terms of solvent, concentrations, temperature, etc.).^[^ ^30^, ^31^, ^32^ ^]^ They can be tailored for gas adsorption and separation processes,^[^ ^33^ ^]^ and for catalysis applications,^[^ ^34^ ^]^ taking advantage of their large surface area and the presence of different metals.^[^ ^35^ ^]^ In addition, MOF attracted a great deal of attention in analytical fields related to the important areas of security, safety, environmental monitoring, and medical diagnostics.^[^ ^36^, ^37^, ^38^, ^39^ ^]^

MOF are particularly attractive for the preconcentration of low molecular weight analytes in conjunction with novel and highly sensitive transducing mechanisms. In this direction, they can be combined with the metallic nanostructures in order to yield SERS substrates empowered with selective trapping VOC analytes at the surface that is optically probed.^[^ ^40^, ^41^, ^42^ ^]^ The potential of MOF‐based SERS substrates for analytical applications has not gone unnoticed and there is a gradually increasing number of reports on the utilization of MOF for (bio)sensing applications^[^ ^43^ ^]^ in the food industry,^[^ ^44^, ^45^ ^]^ environmental monitoring,^[^ ^46^, ^47^ ^]^ and medical diagnostics.^[^ ^48^ ^]^

**Figure 1:**
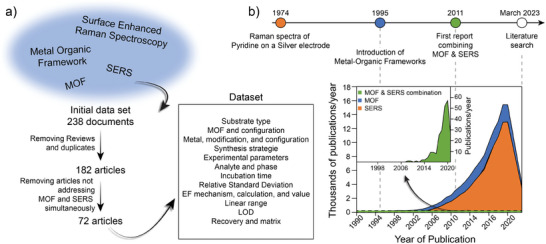
a) Schematic representation of dataset construction from search of specific keywords on Web of Science, curating, and data extraction process. b) Number of publications (not cumulative) on SERS, MOF, and the combination of both MOF and SERS together with milestone events. Full dataset is openly available at a repository.^[^ ^49^ ^]^

This review concerns the current state and potential future trends in the analysis of VOC by the use of SERS in combination with analyte preconcentration enabled by tailored MOF. Both SERS and MOF represent established research fields that have already received enormous attention from the scientific community. Interestingly, MOF‐based SERS sensors gained momentum only recently, and the curation process of literature search to generate a comprehensive dataset^[^ ^49^ ^]^ for the literature available (schematized in **Figure** **1**a) indicates that the focus of the scientific community is rapidly shifting to this emerging research area. An interesting point can be made from the publications‐per‐year rate for the different disciplines (not cumulative). Since the first report of SERS nearly 50 years ago^[^ ^50^ ^]^ and the introduction of MOF as a field almost 30 years ago,^[^ ^26^ ^]^ it took (to the best of our knowledge) until 2011 for the first report on MOF‐based SERS to be published (interestingly, detecting VOC).^[^ ^51^ ^]^ Since then, the scientific community has been pushing forward the field, as it can be seen in Figure 1b with steeply rising number of reported works in the last several years. This review provides a short introduction to SERS and the implementation of MOF to the SERS substrate that is followed by the establishing of figures of merit for characterizing their performance. The relevance of appropriate design and integration of both actors into the final substrate (i.e., MOF and plasmonic components) is also discussed across the manuscript as a pivotal point. Afterward, distinct sections are dedicated to applications concerning detection of harmful VOC in industrial and environmental monitoring, analysis of VOC‐based biomarkers in medical diagnostics, and potential future applications on sensing of VOC for aroma and flavor profiling.

## 2 General Overview

This section is devoted to the most important aspects concerning the combination of SERS substrates and MOF materials. Dedicated reviews individually addressing these topics can be found in the literature, and they are suggested to the reader.^[^ ^20^, ^52^, ^53^, ^54^, ^55^, ^56^ ^]^

### 2.1 Surface‐Enhanced Raman Scattering (SERS)

**Figure 2:**
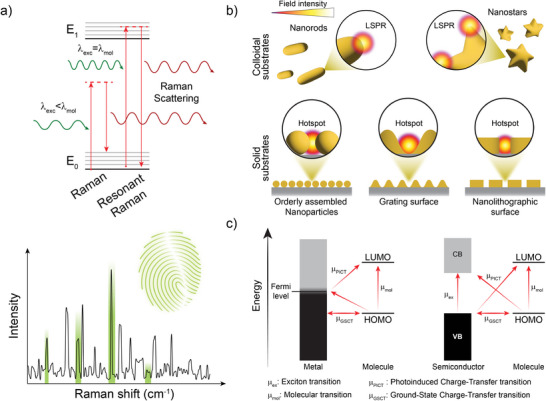
a) Schematics of the Raman scattering process. b) Examples of colloidal or solid substrates toward Surface‐Enhanced Raman Scattering (SERS) generated by (top) colloidal entities or (bottom) nanofabrication of plasmonic surfaces. c) Comparison of possible excitations (resonances) in a (left) metal‐molecule and (right) semiconductor‐molecule charge‐transfer complex for Chemical enhancement Mechanism.

Light couples with molecules via their polarizability leading to either elastic or inelastic scattering. One of the possible inelastic scattering routes is Raman scattering. It is a weak process that is roughly 10^6^ times less probable than elastic Rayleigh scattering and occurs at narrow wavelength bands that are shifted by discrete energy levels from that of the impinging photon. These shifts are characteristic of the chemical structure of the molecules and thus spectral analysis of Raman‐scattered light can be used as a powerful identification tool (see **Figure** **2**a).^[^ ^57^ ^]^ Raman scattering is commonly described based on the interaction with vibrational states of the molecule, and it is associated with absorbing (or releasing) vibrational energy in Stokes (or anti‐Stokes) series of bands. If the wavelength of impinging light matches the energy of the electronic absorption of the molecule, *resonant* Raman scattering can occur with greatly increased efficiency. An alternative means for (arguably) more versatile enhancement of inherently weak Raman scattering is pursued by using metallic nanostructures. They exhibit collective oscillations of charge density coupled with respective electromagnetic fields that are referred to as surface plasmons. Their resonant optical excitation allows for tight confinement of electromagnetic field and leads to a strong enhancement of its intensity. Plasmonic metallic nanostructures can be formed on continuous metallic films supporting propagating surface plasmons (PSPs) or by metallic nanoparticles with sharp edges, tips, or crevices generating the so‐called localized surface plasmons (LSPs).^[^ ^58^ ^]^ When a molecule is placed within the intense surface plasmon field, the yield in Raman scattering is greatly enhanced as it scales with the electromagnetic field amplitude to the power of four. This effect is the basis for Electromagnetic enhancement Mechanism (EM), and it was reported to allow for extraordinarily high enhancement factors of 10^6^–10^12^.^[^ ^59^, ^60^ ^]^ The rich diversity of plasmonic structures that can be obtained either by chemical synthesis of colloids, by their self‐assembly to ordered structures or by nanofabrication techniques, allows for the precise engineering and control of wavelength bands where the plasmonic field intensity enhancement occurs. It also allows for the design of specific locations where the electromagnetic field enhancement peaks that are referred to as plasmonic “hotspots” (see Figure 2b).

Besides the EM contribution to the enhancement mechanism behind SERS, the Chemical enhancement Mechanism (CM) also affects Raman scattering efficiency. It relates to the formation of charge‐transfer complexes between the target molecule and the metallic surface.^[^ ^61^ ^]^ As shown in Figure 2c, there is a whole set of energy transitions possible, either from the metal to the molecule or vice versa (including intrinsic transitions within the molecule related to already established resonant Raman scattering). SERS charge‐transfer in metal‐molecule complexes is maximized when the metal state involved is at the Fermi energy.^[^ ^57^ ^]^ Ground‐state charge‐transfer (µ_GSCT_) is also possible when the energy levels of the SERS substrate and the analyte are aligned. When the energy of the excitation wavelength matches the energy difference for a charge transfer (CT) between the metal and the molecule in any direction, photoinduced charge transfer (µ_PICT_) processes take place. This µ_PICT_ process is of significant importance, and we will come back to it later. CM is also possible with semiconductor‐based SERS substrates.^[^ ^62^ ^]^ Molecular resonance transitions (µ_mol_) are also possible, as well as exciton resonances (µ_ex_), and both contribute to the CM (excitons are hole‐electron pairs generated in solids by optical excitation). Orchestrating these transitions is one of the most critical aspects for SERS‐substrates design. It is important to highlight here that the classification of the enhancement mechanisms in terms of EM or CM is a simplification and these processes are related to each other. SERS should be regarded as a concomitance of all these effects, affecting to a greater or lesser extent the overall enhancement according to the wavelength employed and the analyte‐surface interaction.

The strong enhancement factors accessible for SERS through the combined EM and CM contributions requires the presence of the probed molecule at the close proximity to the metallic surface (<3–5 nm) given the rapid decay of the LSP field with distance from the substrate (for EM) and the need to establish charge‐transfer complexes between the analyte and the SERS substrate (for CM). This is particularly challenging for the detection of VOC given their volatile nature and typically low adsorption capacity on metallic surfaces. Fairly recently, a comprehensive review has been published highlighting the main strategies in tackling the issue of delivering of the analyte toward the hotspot generated at the surface of SERS substrates.^[^ ^63^ ^]^ Along these lines, chemical strategies, i.e., grafting of recognition elements that specifically capture the analyte(s) at the plasmonic hotspot surface are possible. Following complementary physical strategies, research in the engineering of the wettability of the surface for drying the analyzed samples on SERS substrates and the introduction of a porous environment that allows for capturing the target analyte (e.g., from the gas phase) to increase its local concentration within the hotspot decay are pursued. At this point, the versatility of Metal–Organic Frameworks (MOF) makes them particularly attractive in developing a new generation of SERS substrates.

### 2.2 Metal–Organic Frameworks (MOF)

**Figure 3:**
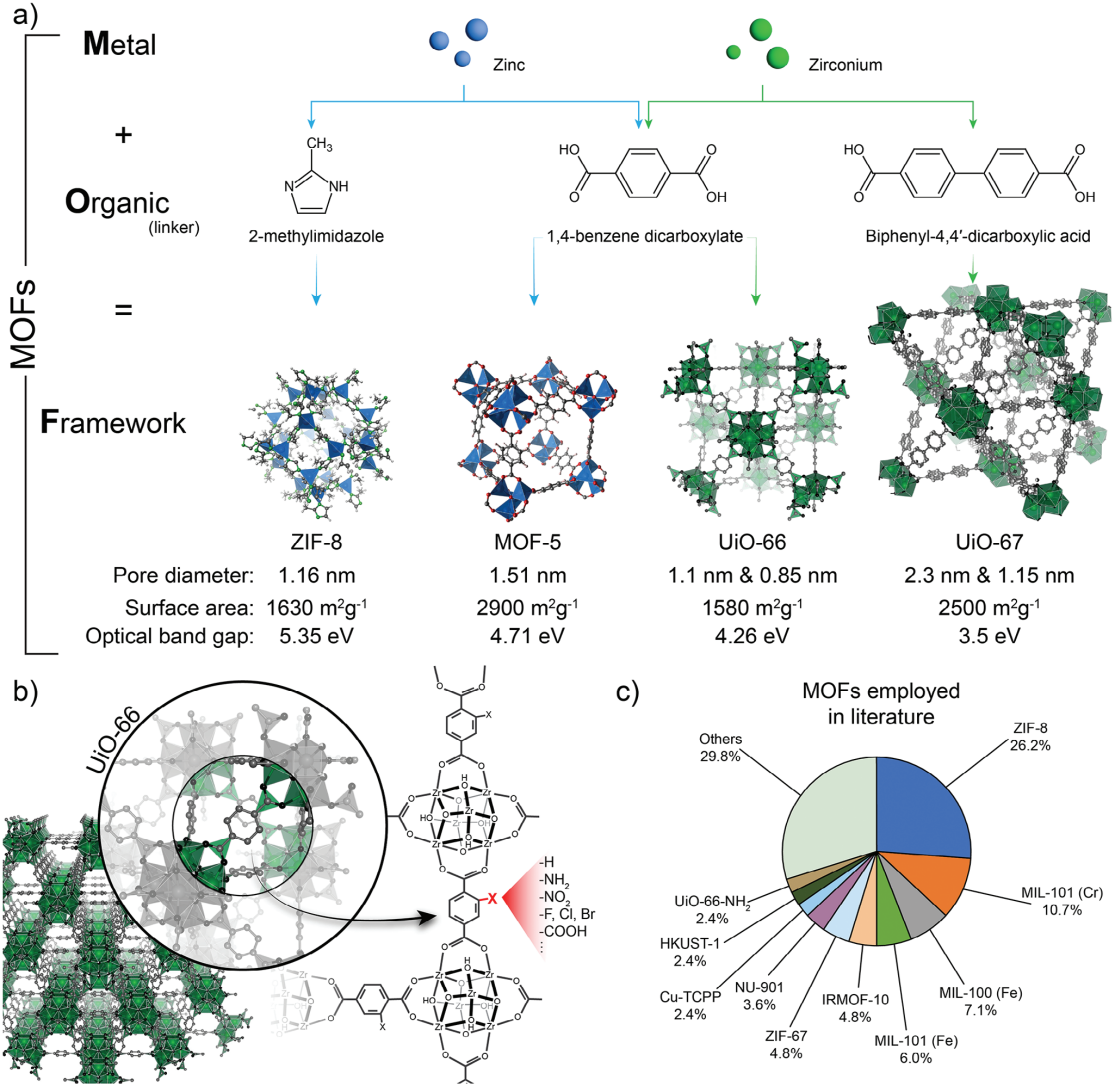
a) Schematic representation of MOF, comparing their different building blocks and possible combinations for developing diverse structures, together with some relevant features. b) Schematic representation of chemical modification of MOF using UiO‐66 as example, where BDC linker can be modified with different dangling groups. c) Top‐10 of most employed MOF for SERS applications, as reflected in the dataset generated for this work.^[^ ^49^ ^]^

MOF are a prominent class of materials^[^ ^26^ ^]^ with metallic centers (which could be either single cations or oxo‐clusters) that are coordinated by organic molecules called *linkers*. Such linkers are multidentate and can therefore extend a network of coordinative bonds where each coordination point interacts with a different metallic center. These perfectly arranged coordinative networks can be crystalline and create closed structures with voids, called micropores (as per IUPAC recommendation, pores with dimensions under 2 nm are called *micropores*; between 2–50 nm *mesopores*; above 50 nm *macropores*). The beauty of these materials is that by changing the metallic or the organic counterpart, one can tune the nature of the generated porous structure. As it can be seen in **Figure** **3**a, the use of zinc and 2‐methylimidazole will endow a MOF called ZIF‐8, with micropores of 1.16 nm and a surface area of 1630 m^2^ g^−1^.^[^ ^64^ ^]^ However, if the linker is replaced by 1,4‐benzenedicarboxylate (BDC) and the synthetic conditions are tuned accordingly, MOF‐5 will be obtained, which features larger micropores (1.51 nm) and larger surface area (up to 2900 m^2^ g^−1^).^[^ ^65^ ^]^ If the same linker is used (BDC) and now zirconium is employed as metallic building block, the MOF UiO‐66 will be obtained, which features two micropore domains, the larger one being 1.1 nm and the smaller one 0.85 nm, with a total surface area of 1580 m^2^ g^−1^.^[^ ^66^ ^]^ By using the same building blocks and changing the linker to biphenyl‐4,4′‐dicarboxylic acid (about double the length of BDC), the MOF UiO‐67 with wider 2.3 and 1.15 nm pores is prepared. And these are just a few examples out of the enormous number of possible combinations. This versatility in terms of chemistry and structure translates also into the conditions that can/must be employed for the synthesis of MOF and their integration in different platforms and devices.^[^ ^38^, ^67^ ^]^ Detailed description of the MOF synthetic approaches is beyond the scope of this review as excellent dedicated works can be found in the literature.^[^ ^68^, ^69^, ^70^ ^]^ However, selected examples of the synthetic approach for the preparation of MOF will be provided to highlight the key principles behind the design of the substrates.

The enormous versatility of MOF in terms of both structure and composition allows for an almost *ad‐hoc* design of their optical properties as well.^[^ ^71^, ^72^, ^73^ ^]^ However, it also imposes a challenge to determining and predicting those properties, since these materials possess nature between ordered extended solids, and molecular systems with highly‐localized bonding interactions.^[^ ^74^, ^75^ ^]^ Perhaps the most relevant parameter when investigating the combination of MOF and SERS (as it will be discussed in the next section) is the optical bandgap (E_opt_). Given the ambivalent character of MOF (whether they can be treated as molecules or extended solids), it is worth summarizing the differences between the reported optical parameters that can be found in literature.^[^ ^76^ ^]^ Shortly, when addressing molecules, the E_opt_ refers to the lowest energy required for an electronic transition accessible via absorption of a single photon, and it is significantly below the fundamental gap (difference between ionization potential and electron affinity). This is contrary to the ionized states due to the electron‐hole pair binding energy (since they remain electrostatically bound). As the molecular size increases when turning small molecules into polymers, electronic bands are formed. Similar to classical semiconductors, this new band structure will present a valence and conduction band. However, the degree of delocalization of the electron wavefunctions will be less pronounced than for ordered structures, relying on the balance between electronic coupling and disorder in the structure. The band structure then allows defining a bandgap between the conduction and valence bands. Being the equivalent to the molecular fundamental gap for materials, it must not be mistaken for the optical gap. Examples of optical gaps are included in Figure 3a, and they refer to the lowest energy of molecular state transition facilitated by a photon absorption. Again, MOF will then lay somewhere in between these two worlds, with their optical properties explained and discussed either in terms of the HOMO‐LUMO energy difference, or in terms of band structure.^[^ ^73^, ^75^ ^]^

The chemical versatility of MOF relies also on the use of linkers within the same family but featuring different dangling groups. This is nicely explored in literature for the case of UiO‐66 for example. Here, the use of 1,4‐benzendicarboxylic acid (BDC) is employed as linker to obtain the “native” UiO‐66.^[^ ^77^ ^]^ However, as schematized in Figure 3b, mono‐ and disubstituted linkers can be employed, featuring ‐NH_2_, ‐NO_2_, ‐COOH groups among others, as well as different halogen heteroatoms.^[^ ^66^, ^78^, ^79^, ^80^ ^]^ All of these chemical modifications alter not only the pore chemistry but also its accessibility, since the dangling groups can occupy either the pore entrance or the pore volume. In addition, likers can also be used in different proportions, as shown for the UiO‐66‐NH_2_ derivative, with relatively straightforward control on the final composition of the MOF.^[^ ^81^, ^82^ ^]^ However, special care must be taken when exploring the functionalization of the structure, since it can also affect the stability of the MOF. For example, the use of 2‐sulfonylterephthalic acid (BDC‐SO_3_H) as linker creates a structure that collapses upon removal of the residual solvent from the pores, because of the high charge state. The collapse can be avoided if the content of this linker is reduced below 40%.^[^ ^83^ ^]^

Despite the vast array of MOF structures and the virtually limitless modifications possible, our analysis of the dataset generated for this review^[^ ^49^ ^]^ reveals that up to now 38 different MOF were explored as/in SERS substrates, and only 10 of them served in 70% of the reported works, as summarized in Figure 3c. The most frequently used MOF appears to be ZIF‐8, probably due to its straightforward (and versatile) synthesis, robustness, and hydrophobic microporosity, which makes it ideal for the adsorption of non‐polar molecules. This importantly highlights the incipience of the field and the still unexplored potential that it represents.

### 2.3 SERS Substrates with Metal–Organic Frameworks

The sharp size exclusion endowed to MOF by their narrow porosity together with their controlled pore chemistry, make these materials excellent candidates for selective VOC capture and preconcentration.^[^ ^42^ ^]^ Importantly, the thickness of MOF films or size of MOF particles can be adjusted to match the decay length of the confined electromagnetic field of LSP. When deployed on SERS substrates, this property allows to optimize the performance of the combined metallic nanostructures and MOF in terms of EM contribution to enhancement of Raman scattering. Furthermore, MOF can be treated as semiconductor‐like light harvesters with reprogrammable optical gaps thanks to their chemical and structural diversity, thus tapping on efficient CM contribution.^[^ ^41^, ^73^, ^75^ ^]^

**Figure 4:**
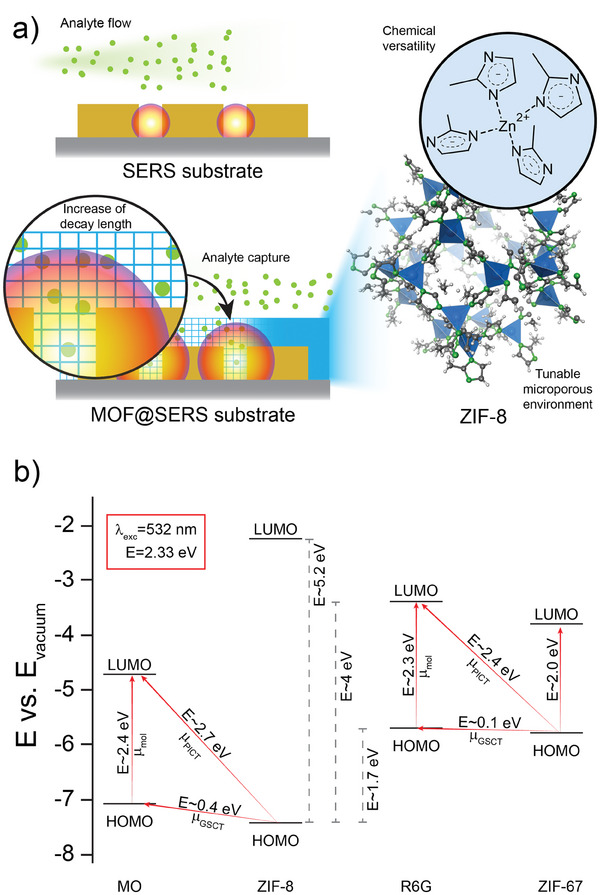
a) Schematic representation of the role of MOF on SERS substrates. b) Energy band matching for ZIF‐8 and ZIF‐67 MOF and two analytes: Rhodamine 6G (R6G) and Methyl Orange (MO) considering an excitation wavelength of 532 nm (values reported by Sun et al.^[^ ^41^ ^]^).

Research on SERS substrates with carefully tuned MOF is a promising field that has been progressing over the last decade.^[^ ^84^, ^85^, ^86^, ^87^ ^]^ In general, there are several characteristics that MOF offer in SERS measurements: i) MOF natural porosity allows to accumulate the target analytes close enough to the SERS substrate so they can be probed by the confined LSP field, as schematized in **Figure** **4**a.^[^ ^88^ ^]^ ii) MOF can act as a molecular sieve functioning for size‐exclusion and provides tunable polar character in the vicinity of the metallic surface. Therefore, for example, moisture‐saturation of active sites can be avoided and/or controlled.^[^ ^89^ ^]^ iii) Non‐specific interactions and secondary reactions such as oxidation or poisoning of metallic surfaces will affect SERS response. The chemical, thermal, and mechanical stability of MOF helps to prevent those effects.^[^ ^90^ ^]^ iv) Enhancing the EM contribution to SERS by the confinement of LSP field though the increased refractive index of MOF (Figure 4a). v) By choosing the appropriate MOF‐analyte combinations that allow coupling in terms of optical gaps matching HOMO‐LUMO levels of the analyte results into a CM with high enhancement factors, close to those reported for EM.^[^ ^41^ ^]^

As an example of the latter, energy band matching diagrams for ZIF‐8 and two Raman‐active molecules – Rhodamine 6G (R6G) and Methyl Orange (MO) are shown in Figure 4b.^[^ ^41^ ^]^ Assuming a 532 nm laser (2.33 eV) is employed as light source for Raman scattering on MO, µ_PICT_ transition from HOMO level in ZIF‐8 can be expected to take place toward the LUMO level of MO. Additionally, µ_GSCT_ transition can also occur given the small difference between the energy of both HOMO levels as well as molecular resonance of MO. Interestingly, these interactions would have negligible effect to the enhancement of Raman signal for R6G, since the energy mismatch between ZIF‐8 and R6G HOMO‐LUMO levels. Nonetheless, this can be reverted if, for example, ZIF‐67 is employed instead of ZIF‐8. ZIF‐67 is an isomorph of ZIF‐8 with Zn atoms being replaced by Co and it exhibits its HOMO level at −5.80 eV and LUMO level at −3.81 eV, representing an optical band gap of 1.99 eV. Now, ZIF‐67 can actively couple with R6G, but will be inactive for MO. All the different aspects highlighted before contributing to the overall enhancement of the Raman signal in SERS and pairs MOF‐analyte can be designed *ad‐hoc* for specific applications.

In Section 2.1, the characteristics of metallic nanostructures supporting LSPs influencing SERS were discussed, and Section 2.2 presented the optical properties of MOF that can/must be tuned in a way that maximizes the SERS performance by matching the optical gap with HOMO‐LUMO levels of the analyte. There is an additional level of complexity that must be addressed now, that arises from merging of the metallic nanostructures with MOF, since the optical properties of such composite materials differ from those of the individual constituents.

**Figure 5:**
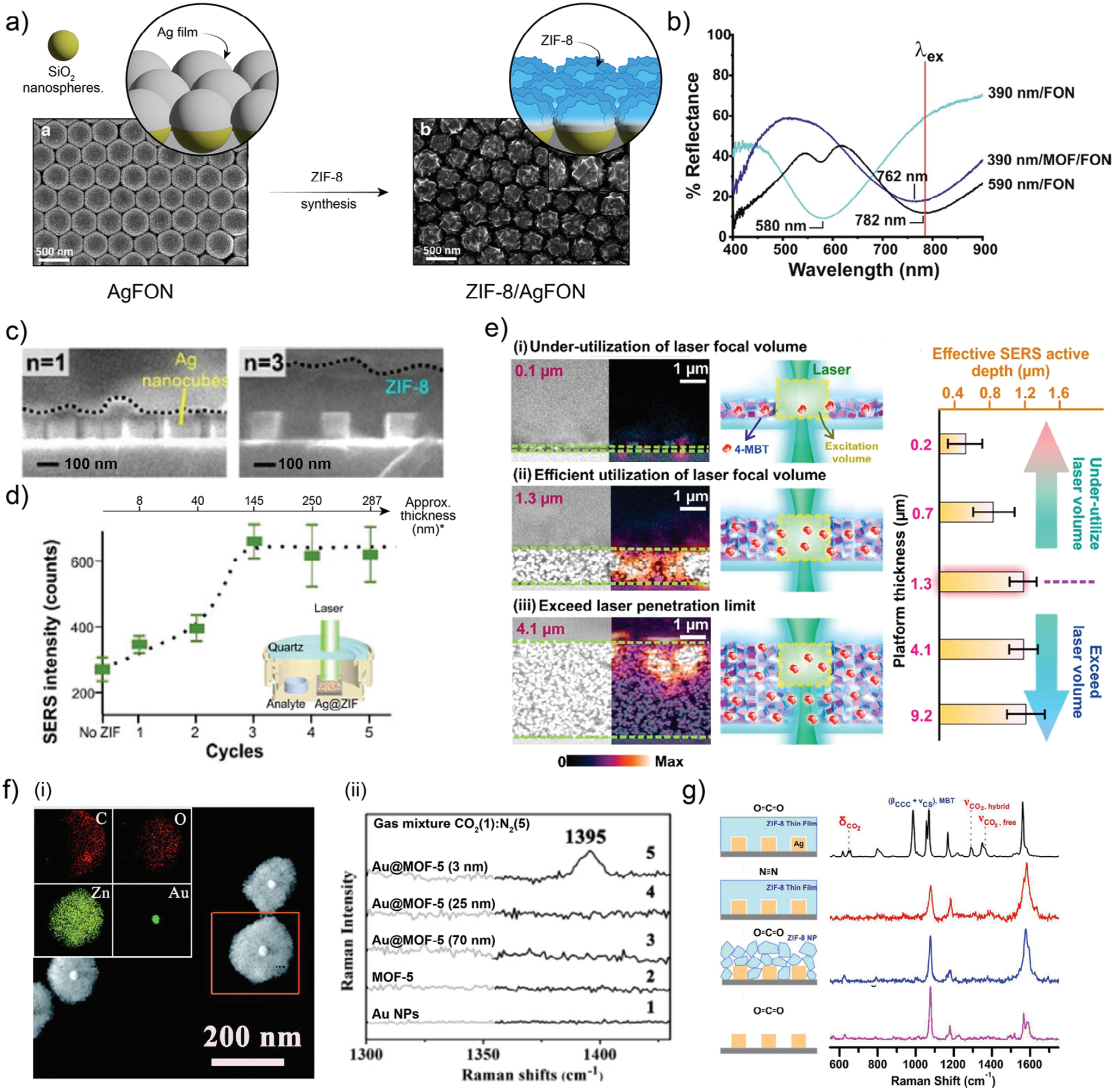
a) SEM images and schematics of the preparation of “films‐over‐nanospheres” (FON) based on ZIF‐8‐coated AgFON. b) Optimization of the FON structure for the LSPR to match laser excitation. Reproduced with permission from Kreno et al.^[^ ^88^ ^]^ Copyright 2014, The Royal Society of Chemistry. c) Profile SEM images of Ag nanocubes assembled by Langmuir–Blodgett covered with ZIF‐8 films with different thicknesses. d) Optimization of ZIF‐8 thickness (as per growth cycles) of the SERS intensity for the detection of gaseous 4‐methylbenzenethiol (4‐MBT). Thickness estimated from reported data. Reproduced with permission from Koh et al.^[^ ^94^ ^]^ Copyright 2018, The Royal Society of Chemistry. e) SEM and hyperspectral images and schemes for thickness optimization of the SERS substrates. Reproduced with permission from Phan‐Quang et al.^[^ ^95^ ^]^ Copyright 2019, American Chemical Society. f) ‐ (i) Single core‐shell Au@MOF‐5 NPs with controllable shell thicknesses. (ii) CO_2_ detection capacity for the different MOF‐5 shell thicknesses. Reproduced with permission from He et al.^[^ ^96^ ^]^ Copyright 2013, Wiley‐VCH Verlag GmbH & Co. KGaA. g) SERS response toward CO_2_ detection of Ag nanocubes encapsulated within ZIF‐8 and the different control experiments employing just a N_2_ flow, pre‐synthesized ZIF‐8 nanoparticles as modification for the Ag nanocubes and bare nanocubes assembly. Reproduced with permission from Lee et al.^[^ ^100^ ^]^ Copyright 2017, American Chemical Society.

This was shown in the study of Kreno et al.,^[^ ^88^ ^]^ where a Film‐Over‐Nanosphere (FON) substrate was developed for detecting VOC including benzene, nitrobenzene, toluene, pyridine, and 2,6‐di‐tert‐butylpyridine. The preparation of the FON structure, illustrated in **Figure** **5**a, involves the self‐assembly of SiO_2_ nanospheres into a hexagonal pattern followed by deposition of Ag thin film by vacuum thermal evaporation. The obtained structure exhibits an LSPR at a resonant wavelength controlled by the nanosphere diameter (Figure 5b). ZIF‐8 was then used to modify the plasmonic substrate, for which it was exposed for 30 min to methanolic solutions of zinc and 2‐methylimidazole mixed in a 1:2 ratio. These steps are typically considered as a single growth cycle, and by its repeating, it allows for further increase in the thickness of the film. The whole approach for the growth of the MOF phase is called Liquid‐Phase Epitaxial (LPE) growth.^[^ ^91^, ^92^ ^]^ After the growth of the MOF phase on the FON substrate, the authors observed a red shift of nearly 30% in the resonant wavelength. This significant shift highlights the intricate interplay between the plasmonic element and the MOF and underscores the importance of not only considering the individual optical response and performance of these components but also comprehending their complex interactions and synergistic effects. Once this interplay is understood, there also arises the question of the required amount of each component on the substrate that is needed to maximize the performance of the platform. It is known that MOF films present the so‐called surface barrier phenomena, which hinders the adsorption of analytes once the outer part of the film is exposed to the sample.^[^ ^93^ ^]^ Unnecessarily thick MOF layers will then prolong the incubation time needed to reach the maximum signal. In addition, analyte molecules adsorbed beyond the decay length of the resonantly excited LSPs will not contribute to the SERS signal. Because of this, thickness becomes a critical parameter, which was for instance explored in the work of Koh et al.^[^ ^94^ ^]^ They assembled Ag nanocubes on a solid substrate by using the Langmuir–Blodgett technique and covered them by a thin ZIF‐8 film grown by the LPE method previously described. The thickness of ZIF‐8 overlayer was controlled between 8 and 287 nm by number of LPE cycles between 1 to 5, see Figure 5c. This MOF‐based SERS substrate was tested for the analysis of 4‐methylbenzenethiol (4‐MBT) and the obtained dependence of SERS signal on the number LPE cycles is seen in Figure 5d. The signal strongly increases with MOF deposition cycles until the 3rd cycle, after which it levels. The obtained SERS signal intensity is a convolution of several factors including the thickness and structure of the porous MOF material that affects the loading with the target analyte. In addition, the plasmonic performance of the incorporated metallic nanostructures are also important, since they determine the extent of the electromagnetic field intensity enhancement contributing to the EM mechanism. The authors examined this aspect by altering the packing density of the Ag nanocubes by adjusting the surface pressure in the Langmuir–Blodgett. They have found that SERS intensity increases with surface pressure, facilitating the enhanced plasmonic coupling within the Ag nanocubes, which translated into an increase of the analytical enhancement factor (AEF, see Section 2.4) by one order of magnitude.

One more step that can be taken for optimizing the performance of MOF‐based SERS substrates is based on matching the focal volume of probing laser beam with the thickness of the prepared structure. Phan‐Quang et al., explored this dependence by using a multilayer of Ag nanocubes coated with ZIF‐8 were prepared by drop‐casting.^[^ ^95^ ^]^ In this work SEM topography measurements were combined with SERS hyperspectral imaging of infiltrated 4‐MBT analyte. As it can be seen in Figure 5e, thin assemblies under‐utilize the focal volume of the laser beam, while thicknesses >1 µm no longer contribute to the signal output. Now, even when the full focal volume of the laser is being employed, the distribution of metallic nanostructures in it is also important. The authors also show that when multilayers of ZIF‐8‐coated Ag nanocubes are prepared with thicknesses matching the size of the laser beam focal volume, the whole assembly contributes to the output signal. However, if the MOF is now localized just at the topmost part of an equally thick assembly of Ag nanocubes, the signal originates just from the top region, since the ZIF‐8 layer is responsible for capturing and preconcentrating the analyte within the LSP decay length of the plasmonic elements. This becomes even more evident when MOF is not present at all, and thus just a little amount of analyte interacts with the Ag nanocubes.

At this point, it becomes clear that in order to develop an efficient MOF‐based SERS detection platform, the structure of both the plasmonic metallic part and the MOF must be mutually tailored. For example, the mere design of the MOF constituent for fast diffusion of the analyte through its porosity, is not sufficient if the plasmonic characteristics of the caped metallic nanostructure are detuned from the chosen wavelength. This important interplay was nicely demonstrated in the work of He et al.^[^ ^96^ ^]^ Here, the authors synthesized single‐core Au@MOF‐5 NPs by the one‐pot approach: all reactants were mixed together (Au NPs precursors, PVP for their later capping and stabilization, and Zn(NO_3_)_2_ and terephthalic acid, the MOF precursors). Given the time differences in the nucleation and growth processes, Au NPs were first grown withing this mixture, and then they were modified by PVP, which stabilizes them. Later, MOF‐5 nucleation started from the surface of the Au NPs, developing well dispersed single‐core‐shell Au NP, as seen in Figure 5f(i). By controlling the synthetic conditions, Au cores with diameters of 54 ± 7, 38 ± 7, and 30 ± 7 nm were obtained, and the MOF shell thickness varied for each case: 3.2 ± 0.5, 25 ± 4, 69 ± 12 nm respectively. These core‐shell structures were then employed for the detection of CO_2_, capable of diffusing across the MOF porous structure. As shown in Figure 5f(ii), NPs with 54 ± 7 nm Au cores and 3.2 ± 0.5 nm thick MOF‐5 shells were the only ones that presented activity toward the detection of CO_2_ even though all of the composites were crystalline and porous. This result makes clear the need for optimizing the complete structure of the MOF‐SERS substrate. It is not only the contribution of the MOF‐5 shell that has an impact on the performance, but also the size of the plasmonic NPs needs to be adjusted respectively.

As a final remark, let us point out that MOF are not perfect defect‐free crystals. When MOF layers are synthesized, the growing fronts inevitably coalesce leaving empty space with larger dimensions than the intrinsic (micro)porosity. This additional porosity is typically called Constructional Porosity, and it was proven to be the main factor controlling diffusional processes through MOF.^[^ ^89^, ^97^, ^98^, ^99^ ^]^ This phenomenon adds one additional level of complexity to structure‐performance relation in MOF‐based SERS substrates. For example, Lee et al. reported on molecular‐scale interfacial cavities (i.e., constructional porosity) between the used Ag nanocubes and the ZIF‐8 film grown by LPE method (Ag@ZIF‐8), which enables preconcentrating of target CO_2_ analyte nearby the surface of the Ag nanocubes with no chemical bonding taking place.^[^ ^100^ ^]^ As it can be seen in Figure 5g, bare Ag nanostructure modified with 4‐MBT does not respond to CO_2_ contacted to its surface. However, when it is capped with ZIF‐8, CO_2_ exposure leads to the appearance of respective Raman scattering peaks. Importantly, when pre‐synthesized ZIF‐8 particles are cast on the Ag‐MBT array, the cavities generated are larger, and no preconcentration of CO_2_ takes place within the LSP decay length.

To summarize, the structural and chemical tunability of MOFs, together with the fine control over their thickness (either as films or shells surrounding nanoparticles) help to maximize the electromagnetic and chemical mechanisms contribution to the SERS signal. Matching the decay length of the LSP, the resonant wavelength of the substrate, and enabling the formation of charge‐transfer complexes between the MOF and the analyte, is necessary to maximize the performance of the platform. All the above implies that systematic and robust design and analysis of the substrate properties must be done, since the emerging properties of the MOF‐based SERS substrate such as shifts in resonant wavelength, or the presence of constructional porosity, will differ from the individual constituents, and in most cases determine the final response of the system.

### 2.4 MOF‐Based SERS Detection Formats and Performance Characteristics

The richness of MOF chemical and structural properties has stimulated the development of a vast quantity of MOF‐based SERS detection platforms. As revealed in our dataset,^[^ ^49^ ^]^ so far there are 38 different MOF serving for detecting of 103 analytes. Approximately 30% of the detected analytes are dyes and this prevalence can be attributed to the necessity of detecting industrial dyes in watercourses^[^ ^101^ ^]^ and also to their common use as benchmarking compounds for Enhancement Factor evaluation (see below).^[^ ^53^ ^]^ While the complete dataset has been made available, **Table** **1** presents select examples, illustrating the use of MOF for SERS measurements with and without (plasmonic) metallic nanostructure component. MOF versatility extends to pore chemistry, accessibility, and flexibility, emphasizing their potential in VOC detection. Combining MOF with SERS substrates offers a promising path for advancing VOC detection, as showcased in Table 1, highlighting the varied applications of these systems.

**Table 1:** Selected examples from dataset for MOF – analytes combinations in SERS‐based detection of VOC. For pore diameters, the larger one (if more than one) is reported. When no value i,s reported, it is assumed to be the same as the closest MOF in the family, e.g., MIL‐100 (Fe) similar than MIL‐100 (Al). QDs: Quantum Dots. All examples and references can be found in the dataset openly available in a repository.^[^ ^49^ ^]^

MOF	Larger Pore diameter [nm]	Plasmonic Metal	Analyte(s)
HKUST‐1	0.9	Au, Ag	4‐aminothiophenol, 4‐chlorobiphenil, 4‐mercaptobenzoic acid, anthracene, perylene, pyrene
IRMOF‐10	1.3	Au	2,6‐biphenylpyridine, chloroform, CO_2_, DMF, fenitrothion, fumonisin, paraoxon, pyridine, rhodamine 6G, sudan III
MIL‐100 (Al)	2.9	None	methyl orange
MIL‐100 (Cr)		None	methyl orange
MIL‐100 (Cu)		Au	malachite green, methylene blue
MIL‐100 (Fe)		None, Au, Ag	4‐aminothiophenol, 4‐ethylbenzaldehyde, acetone, chloroform, CS_2_, crystal violet, dopamine, isopropanol, malachite green, methylene blue, rhodamine 6G, rhodamine B, 3,3′,5,5′‐tetramethylbenzidine, thiram, toluene
MIL‐101 (Cr)	3.4	None, Au, Ag	4‐aminothiophenol, 4‐mercaptobenzoic acid, 4,4‐bipyridine, alpha‐fetoprotein, benzidine, crystal violet, diphenylamine, enalprilat, folic acid, fosinopril sodium, glucose and lactate, human carboxylesterase 1, isopropanol, methyl orange, nitrofurantio, p‐phenylenediamine, rhodamine 6G, tetrodotoxin
MIL‐101 (Fe)		Au, Ag	cholesterol, creatinine, crystal violet, methenamine, pioglitazone, rhodamine 6G, sildenafil, thiabendazole
MIL‐125 (Ti)	1.25	None	isopropanol, toluene
MIL‐125‐NH_2_ (Ti)		None	toluene
NU‐901	3.1	Au, Ag, Au‐QDs	4′‐mercaptobiphenylcarbonitrile, benzoic acid, biphenyl‐thiol, SO_2_
UiO‐66	1.1	None, Au	2‐amino‐3,4‐dimethyl‐3H‐imidazolquinoline, acetamiprid, diquat, isopropanol, methylene blue, paraquat, rhodamine 6G, toluene
UiO‐66‐NH_2_		Au	malachite green, rhodamine 6G, sudan red 7B, thiabendazole
UiO‐67	2.3	Au	acetamiprid
ZIF‐8	1.16	Au, Ag	1‐butanol, 1‐naphthalenethiol, 2‐chloroethyl ethyl sulfide, 2,6‐di‐tert‐butylpyridine, 3‐ethyl benzaldehyde, 4‐aminothiophenol, 4‐ethylbenzaldehyde, 4‐mercaptobenzoic acid, 4‐methylbenzenethiol, aniline, benzaldehyde, benzene, cadaverine, carbendazim, chlorobenzene, chloroform, CO_2_, crystal violet, dithiohydroquinone, dimethyl methylphosphonate, eriochrome black T, ethylbenzene, eormaldehyde, glutaraldehyde, hexachlorocyclohexane, malachite green, melamine, methylen blue, methyl blue, methyl orange, naphthalene, nitrobenzene, p‐aminothiophenol, p‐hydroxy thiophenol, pyridine, putrescine, rhodamine 6G, thiabendazole, thiram, toluene, toluylene red, xylenol orange

Maximizing SERS signal strength for sensitive analysis of target analytes requires adjusting of instrumental parameters and design of the substrate, as well as considering parameters related to the analyte itself. Different figures of merit were established for quantification of SERS performance utilized in different platforms,^[^ ^53^, ^102^ ^]^ and the so‐called Enhancement Factor (EF) is commonly used for comparing the output signal provided by the SERS substrate to regular Raman scattering:

(1) $$EF=\frac{I_{SERS}}{N_{SERS}}\;\frac{N_{RS}}{I_{RS}}$$

where Raman peak intensity being recorded on the SERS substrate (I_SERS_) from N_SERS_ molecules being probed, are compared against the intensity I_RS_, measured from N_RS_ molecules in Raman scattering configuration without the amplification. It is worth noting that determining the number of molecules being probed is often challenging and leads to uncertainties of determined EF values. There are numerous modifications of EF, and the Analytical Enhancement Factor (AEF) represents one of the most used alternatives. AEF is defined similar to (1), but the number of molecules N is replaced by the nominal concentration of target analyte employed for each measurement. This definition is useful especially when there is no strong interaction between the analyte and the SERS‐active element of the substrate or when using SERS‐active liquids (colloidal suspensions). However, it strongly depends on factors such as surface coverage and sample preparation procedures.^[^ ^103^ ^]^

**Figure 6:**
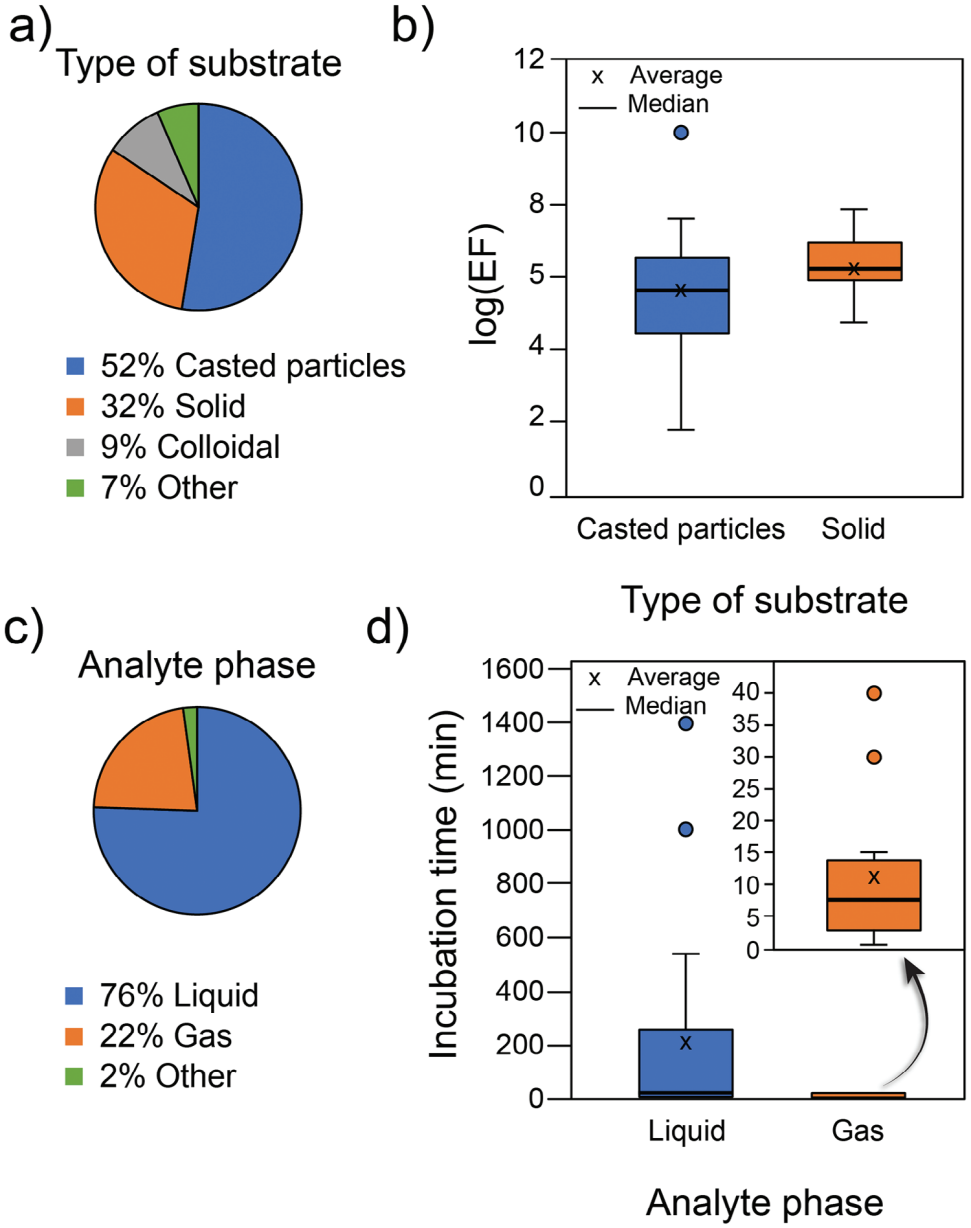
a) Prevalence of each type of substrate employed across literature. b) Enhancement factor as in Equation (1) for the two dominant types of substrates: cast particles and solid substrates. c) Analyte phase prevalence for SERS experiments and the associated d) incubation time (reported as optimized) required to reach constant signal. Data is openly available in a repository.^[^ ^49^ ^]^

As summarized in **Figure** **6**a, casted‐particles substrates represent >50% of the reported experiments for MOF‐based SERS substrates, probably due to the versatility and relatively straightforward synthesis without the need of complex infrastructure. Solid substrates prepared by lithographical means account for 30% of the cases. We have found that among the manuscripts that reported any type of enhancement factor (≈25% of the explored literature) 57% correspond to the EF definition (1) and 43% to the AEF, with no evident link between enhancement factor calculation and substrate type. As shown in Figure 6b, solid substrates allow for a more precise control over the EF obtained compared to casted SERS substrates. If the average of the reported values is compared, EF achievable by solid substrates (1.74 ×·10^6^) are four times higher than the ones obtained from casted particles (4.38·× 10^5^) and present a narrower distribution, probably due to the fine‐tuning of the morphology of the surface by nanolithografic approaches.

When analyzing the performance of a given device toward detecting of specific analyte, time response is extremely relevant. In the case of SERS, where the analyte needs to be docked in close vicinity to the substrate surface, the time response will be highly influenced by the nature of sample. As can be seen in Figure 6c, 76% of the experiments were conducted using liquid samples, as expected from the predominance of cast‐particles substrates, while the rest of the reports employ gaseous samples (22%). The remaining 2% involve other types of samples such as aerosols, pure solids, etc. Through the analysis of average values obtained from box plots (see Figure 6d), it becomes evident that the detection of analytes in the gas phase is faster compared to that in the liquid phase. Specifically, the average detection time for gas‐phase analytes stands at 11 min, while liquid‐phase analytes exhibit a significantly longer average detection time of 264 min. This stark contrast in detection times highlights the advantage of implementing MOF‐based SERS sensors for VOC sensing applications directly from the gas phase.

Regarding the use of SERS for quantification purposes, good analytical practices and different processing methodologies have been proposed. Among them, the need to include validation steps during calibration routines is highlighted. Determining of the Limit of Detection (LOD, defined as a concentration where the sensor response yields three‐times the standard deviation of the blank) can be employed for side‐by‐side comparison since it depends on instrumental factors as well as on the substrate and sample's characteristics. Another important aspect is to consider the use of internal standards. These are Raman reporters that are either placed *ad‐hoc* or part of the structure of the substrate for which the Raman signal (I_standard_) should remain invariable during the course of the experiment. By this strategy, I_standard_ is employed as normalization value and accounts for most of the inter‐substrate variability.^[^ ^53^, ^102^, ^104^ ^]^ Finally, in terms of reproducibility (which should not be mistaken for uniformity), comparisons between batches should be established. These aspects were addressed in a recent interlaboratory study where 15 laboratories, involving 44 researchers from over 11 countries, provided more than 3500 SERS spectra, for which different figures of merit (accuracy, trueness, and precision) were assessed.^[^ ^105^ ^]^ As a result of this analysis, the authors agree that SERS can be employed as a quantitative tool worldwide if well‐defined protocols are implemented.

## 3 Environmental and Industrial Monitoring

The analysis of VOC is highly relevant to environmental monitoring as industrial activity is one of the main anthropogenic sources of VOC. In addition, it is also important for securing indoor air quality since the presence of a wide range of VOC^[^ ^106^, ^107^ ^]^ in households, recreational and workplaces has a direct impact to human health.^[^ ^108^ ^]^ The exposure of human population to VOC is strictly regulated by different organizations. To state several examples, in the United States the Occupational Safety and Health Administration (OSHA) and the National Institute for Occupational Safety and Health (NIOSH) set permissible exposure limits and safe practices related to various chemicals, including VOC. The EU‐OSHA, the European counterpart of OSHA, also provides guidelines and information for European countries. Examples of these guidelines are OSHA Permissible Exposure Limit (PEL, available online at https://www.osha.gov/annotated‐pels) and the NIOSH Recommended exposure limits (NREL, also available online at http://www.cdc.gov/niosh/npg/npg.html). Typical NREL values for VOC are in the range of a few tens to hundreds of ppm. When considering early detection of VOC, however, it would be necessary for the detection platform to provide a response at sub ppm or few ppm units, at least qualitatively. Aromatic, polycyclic, and halogenated hydrocarbons are the most common VOC, given their abundance in industrial activities and use as agrochemicals.^[^ ^109^ ^]^ This fact is reflected in the dataset compiled for this work; excluding dyes (being Rhodamine 6G the most recurrent) and thiols (since they are typically chosen because of their affinity toward the plasmonic surface; the most recurrent was found to be 4‐aminothiophenol), the most explored analyte was found to be toluene, followed by benzidine, while chloroform was the most recurrent halogenated hydrocarbon.

**Figure 7:**
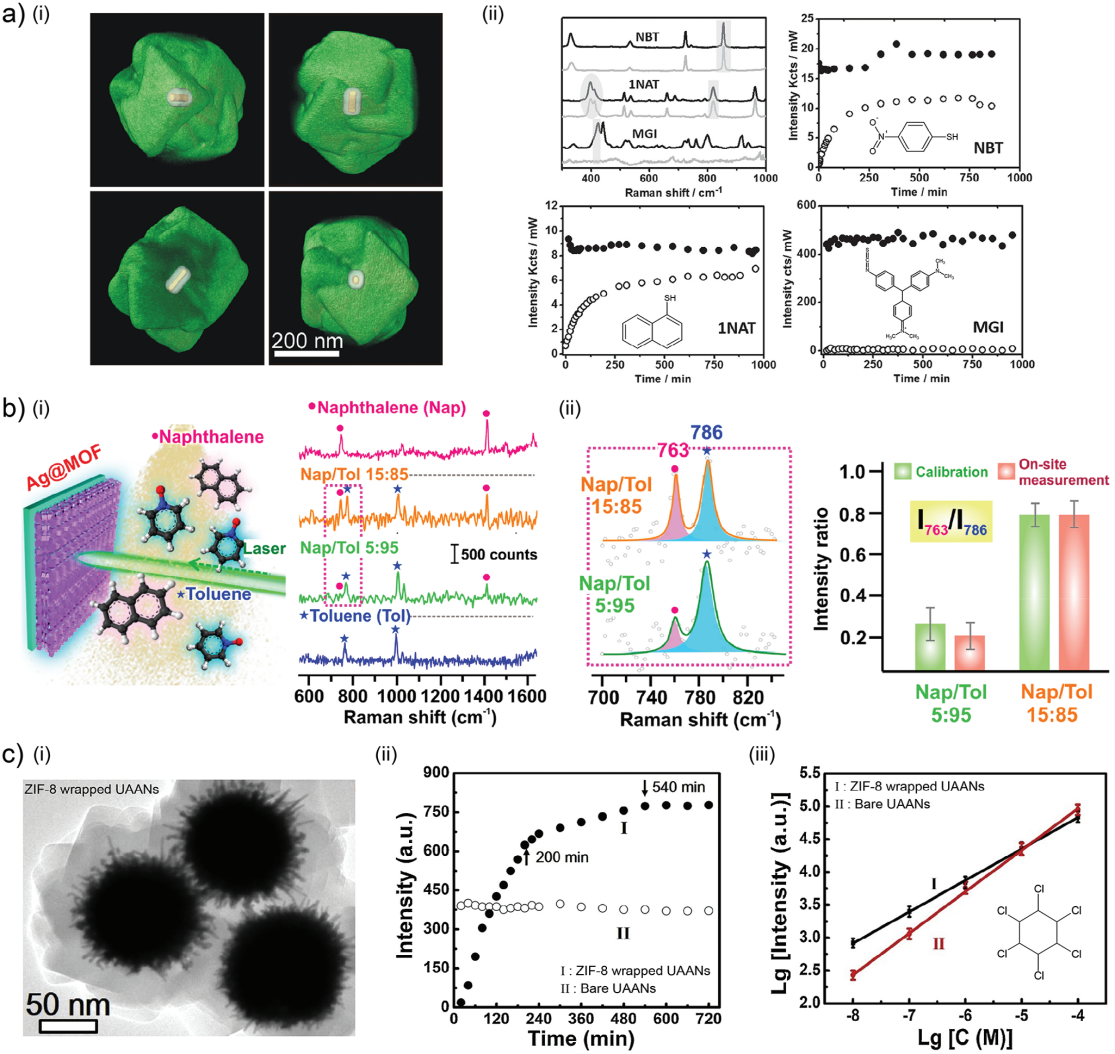
a) ‐ (i) 3D reconstruction of Au/Ag@ZIF‐8 NPs and (ii) SERS spectra (black line: uncoated Au/Ag NPs; grey lines: Au/Ag@ZIF‐8) with their associated temporal evolution for the sensing of 4‐nitrobenzenethiol (NBT), 1‐naphtalenethiol (NAT), and Malachite Green Isothiocyanate (MGI). Reproduced with permission from Zheng et al.^[^ ^115^ ^]^ Copyright 2016, Wiley‐VCH Verlag GmbH & Co. KGaA. b) ‐ (i) Outdoor remote sensing (Raman apparatus – target analyte distance of 2 m) of aerosolized Naphthalene and Toluene mixture, by tracking ii) characteristic peaks from toluene (δ_CH_ at 763 cm^−1^) and naphthalene (δ_CH_ at 786 cm^−1^) intensity ratio. Reproduced with permission from Phan‐Quang et al.^[^ ^95^ ^]^ Copyright 2019, American Chemical Society. c) ‐ (i) TEM image of ZIF‐8 wrapped urchin‐like Au‐Ag nanocrystals (UAANs). (ii) Time evolution for the detection of γ‐hexachlorocyclohexane (γ‐HCH) for bare and wrapped UAANs and (iii) the associated linear range detection. Reproduced with permission from Zhou et al.^[^ ^116^ ^]^ Copyright 2019, Elsevier Inc.

As illustrated in Section 2.3, small molecules such as CO_2_ can easily adsorb within the MOF microporosity. However, as molecular size increases, diffusion through the porous matrix becomes hindered and sieving effects must be taken into account. Among other effects, diffusion becomes relevant for the sensing of aromatic and polycyclic aromatic hydrocarbons (PAH). For example, naphthalene and nitrobenzene represent a serious hazard toward human health and the environment and are widely used in industrial activities. Nitrobenzene is involved in the synthesis of numerous chemicals used as pharmaceuticals and pesticides, it is involved in the petrochemical industry activities, and its emission has even been detected even from small laundry facilities.^[^ ^110^, ^111^, ^112^ ^]^ Naphthalene can also be found in emissions from several industrial activities, ranging from metal production to plastic, pesticides, and surfactants synthesis, and present a severe risk to human health.^[^ ^113^, ^114^ ^]^ Therefore, to develop suitable platforms for fast detection of this family of compounds is of particular interest. Toward this aim, Zheng et al. employed single‐core(metal) – shell(MOF) NPs (**Figure** **7**a(i), for the detection from aqueous solutions of 4‐nitrobenzenethiol (NBT) and 1‐naphtalenethiol (NAT). In their work, they pre‐synthesize Au/Ag nanorods to later encapsulate them in ZIF‐8 by LPE method.^[^ ^115^ ^]^ As shown in Figure 7a(ii), both NBT (5.95 Å kinetic diameter) and NAT (7.20 Å kinetic diameter) can be detected by both bare Ag/Au NPs and Ag/Au@ZIF‐8 particles; however, larger molecules like Malachite Green Isothiocyanate dye (MGI, 13.45 Å kinetic diameter) are not able to reach the surface of the plasmonic core due the sieving effect imposed by the porous framework of ZIF‐8, with 11.6 Å pore diameter. It is worth noticing that access to the ZIF‐8 micropore is restricted by a 3.4 Å rings, which in principle should prevent the access of NAT and NBT as well.^[^ ^64^ ^]^ Short‐lived ligan vacancies (i.e., partial and temporal disruption of the coordinative bonds) and the framework's flexibility were attributed to the ability of the molecules to penetrate into ZIF‐8, although constructional porosity probably played a significant role as well.

Toluene, naphthalene, and derivates can not only be detected in water, but also in the air either as gases or aerosols. Therefore, developing platforms suitable for their detection is also of significant importance. In the work of Phan‐Quang et al., the authors applied the SERS substrate made by multilayered Ag@ZIF‐8 nanocubes (discussed in Section 2.3 of this manuscript) for the stand‐off detection of such VOC.^[^ ^95^ ^]^ A system composed of SERS substrate and spectrometer was deployed outdoor and aerosolized mixtures of Toluene and Naphthalene in different ratios were ejected in the vicinity of the SERS substrate (Figure 7b(i)). By means of a calibration curve established in laboratory conditions and by employing the 4‐MBT as internal standard (Figure 7b(ii)), the authors demonstrated the accurate and parallel quantification of the analytes in different mixtures of Naphthalene/Toluene from the gas phase.

Halogenated hydrocarbons are also of significant interest for environmental monitoring since they are the main components (sometimes even the only one) of agrochemicals, and their detection by MOF‐based SERS platforms has also been explored.^[^ ^116^, ^117^, ^118^ ^]^ Although with lower volatility compared to other VOC, they can be found in the atmosphere as aerosols or in particulate material, and subsequently deposited on the soil, water courses, and even our skin, not to mention their inhalation. An example of this halogenated hydrocarbon is the hexachlorocyclohexane (HCH), a pesticide banned by EPA in 2006 but still found in water courses and soil. This is the case of the work of Zhou et al., where they have explored the development of a core‐shell structure where ≈100 nm urchin‐like Au–Ag alloyed nanocrystals (UAANs) are wrapped by ZIF‐8 with controllable thickness between 10 and 40 nm, as depicted in Figure 7c(i). In their work, the authors have optimized the shell thickness at 20 nm, which presented an enhancement factor of 3.10^7^. Although the porous structure of ZIF‐8 limits the diffusion of the HCH toward the surface of the UAANs, requiring almost 10 h to reach a stable intensity of the Raman signal (Figure 7c(ii)), they show the final intensity to be almost double than for the bare UAANs, reaching a detection limit of 5.10^−9^ m (equivalent to 1.5 ppb).

The authors also show that while ZIF‐8 wrapped UAANs are suitable for trace detection of HCH, thanks to the presence of ZIF‐8 microporosity, sensitivity decreases for higher concentrations, compared with bare UAANs (Figure 7c(iii)). This is ascribed to the same porosity being saturated as bulk concentration of HCH increases. The sieving properties of the platform were explored by showing that a small molecule such as 4‐aminothiophenol can in fact diffuse through the porous framework, while Rhodamine 6G cannot.

**Figure 8:**
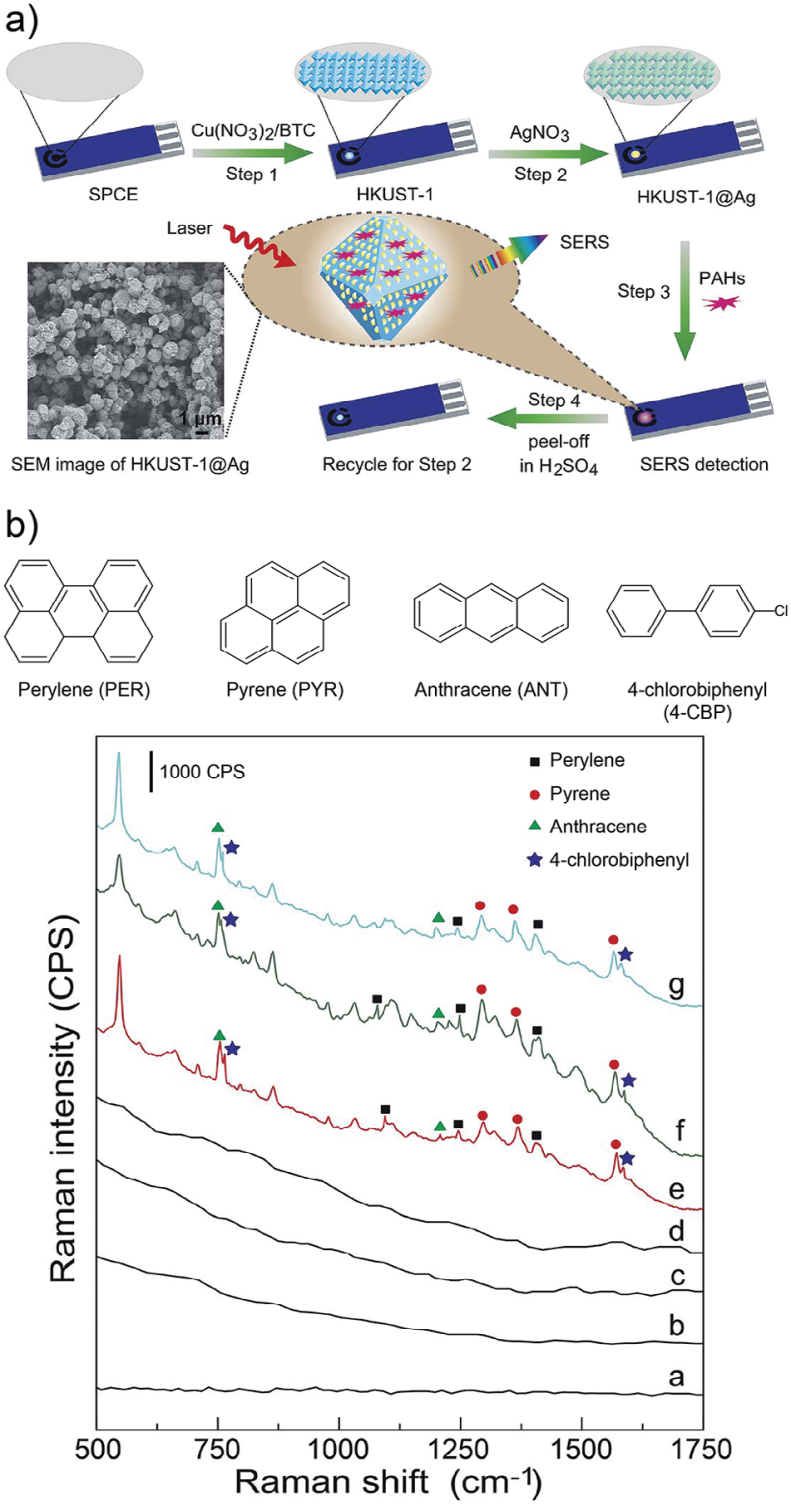
a) Schematic representation for the fabrication and regeneration of HKUST‐1@Ag* SERS substrate for the quali‐quantitative detection of PAHs. b) SERS spectra for the multivariate detection of VOC in (a) blank; b) river water; c) sewage water; d) seawater; e–g) spiked river, sewage, and seawater respectively. *Please note that the nomenclature here is used differently than previously mentioned, being Ag the outer layer of the composite. Reproduced with permission from Li et al.^[^ ^119^ ^]^ Copyright 2019, The Royal Society of Chemistry.

In their work, Li et al. developed a SERS substrate capable of quali‐quantification of PAHs.^[^ ^119^ ^]^ The strategy followed by the authors was based on to using of HKUST‐1 MOF (built from Cu^2+^ and 1,3,5‐benzenetricarboxylic acid ‐H_3_BTC‐) as substrate for the electrodeposition of Ag NPs. The advantage of this structure is that upon use, the Ag can be electrochemically removed, together with the adsorbed analytes, regenerating the HKUST‐1 substrate and reusing it for a new Ag electrodeposition cycle (see **Figure** **8**a). The presence of the MOF allowed the device to be stable in a wide range of pH (from 2 to 12), contrary to the behavior of the bare Ag colloids. The substrates presented also excellent reproducibility, performing with a variation of 8.5% over 50 different substrates. In addition to good stability and reproducibility, the reported SERS substrates were proven suitable for on‐site simultaneous detection (by using portable Raman spectrometer) of different PAHs, namely anthracene (ANT), pyrene (PYR), Perylene (PER), and 4‐chlorobiphenyl (4‐CBP) in river, sewage and seawater samples (Figure 8b) with a performance comparable to GC‐MS. While GC‐MS provided results with a 4.8% RSD, the HKUST‐1‐based SERS substrate did it with an 8.1% RSD, demonstrating the potential of MOF‐based SERS substrates toward the development of suitable detection platforms for VOC.

Despite only a handful of analytes were discussed above, many other examples can be found in the literature. To name a few, the detection of butanol, isopropanol, chloroform, diphenylamine, pyridine derivatives, aldehydes, and acetone was also achieved by MOF‐based SERS substrates.^[^ ^120^, ^121^, ^122^ ^]^ As previously mentioned, different figures of merit are available to benchmark the analytical performance and **Table** **2** provides a short summary of the data compiled in our dataset. LOD reached by the different substrates are summarized and confronted with relevant concentrations in the field of environmental and industrial monitoring.

**Table 2:** Selected examples from dataset for gas‐ and liquid‐phase detection of VOC for Environmental and Industrial monitoring. Relevant concentrations correspond to NIOSH recommended exposure limits (NREL) unless otherwise stated. All examples and references can be found in the dataset openly available in a repository.^[^ ^49^ ^]^

Gas Phase detection					
Analyte	MOF	Metal	Linear range [ppm]	LOD [ppm]	Relevant concentration [ppm]
Acetone	MIL‐100 (Fe)	None	1.25–3.5	20	250
Benzene	ZIF‐8	Ag	–	540	0.1
Cadaverine	ZIF‐8	Au	0.01–10	0.116	2000^a)^
Chloroform	ZIF‐8	Ag	–	0.05	2
Chloroform	MIL‐100 (Fe)	None	2–4	93	2
Isopropanol	MIL‐100 (Fe)	None	–	100	400
Isopropanol	MIL‐125 (Ti)	None	15–1000	120	400
Isopropanol	UiO‐66	None	–	20	400
Isopropanol	MIL‐101 (Cr)	None	–	489	400
Isopropanol	MIL‐100 (0.2Zr‐0.8Fe)	None	–	50	400
Putrescine	ZIF‐8	Au	0.01–10	0.77	2000^a)^
Toluene	MIL‐100 (Fe)	Au	–	5·10^4^	100
Toluene	MIL‐125‐NH_2_ (Ti)	None	–	111	100
Toluene	MIL‐125 (Ti)	None	–	15	100
Toluene	UiO‐66	None	–	15	100
Toluene	MIL‐100 (Fe)	None	0.5–3.5	2.5	100

Liquid Phase detection					
Analyte	MOF	Metal	Linear range [µm]	LOD [nm]	Relevant concentration [nm]
4‐chlorobiphenil	HKUST‐1	Ag	1–1000	5	2.65
Anthracene	HKUST‐1	Ag	1–1000	20	1^b)^
Benzidine	Cu‐TCPP	Au	1 × 10^−6^–100	0.00016	1000^c)^
Benzidine	MIL‐101 (Cr)	Au	–	0.00067	1000^c)^
Perylene	HKUST‐1	Ag	1–1000	3	0.9
p‐Phenylenediamine	MIL‐101 (Cr)	Au	9 × 10^−3^–0.9	0.92	0.9
Pyrene	HKUST‐1	Ag	1–100	0.15	0.5^d)^
Sulfur dioxide	NU‐901	Ag	0.01–100	3.6	80

^a)^ Oral toxicity;

^b)^ NREL for benzanthracene;

^c)^ EC50;

^d)^ NREL for benzopyrene.

Data in Table 2 has been grouped based on the analyte phase. In the case of gas‐phase detection of VOC, both linear ranges and LOD values are more than suitable for detecting the target analytes in concentrations relevant for daily exposure limit, as determined by the NIOSH (time‐weighted‐average occupation exposure limit either for up to 10 h/day or a 40 h workweek). When NIOSH‐Rel values were not available, other indicators were considered, as indicated in the table. The same holds for liquid‐phase detection of analytes. Please note here that concentration in such instances is expressed as micro‐ or nanomolar and not in terms of ppm as in gas‐phase cases. The use of various MOF, such as MIL‐100 (Fe), ZIF‐8, MIL‐125 (Ti), UiO‐66, HKUST‐1, Cu‐TCPP, MIL‐101 (Cr), and NU‐901, combined with different plasmonic nanostructures, demonstrates the possibility of generic analysis of a wide range of analytes. The linear ranges, limit of detection values, and relevant concentrations vary depending on the specific combination of MOF, metal, and analyte. An interesting point comes forth when analyzing the data in Table 2 where no metal is present, as in the case of acetone, chloroform, isopropanol, and toluene for gas‐phase detection. In all these cases, it can be seen that the chemical enhancement mechanism provided by the MOF was sufficient to develop SERS substrates capable of detecting the VOC at concentrations far below those stipulated by regulatory bodies (NIOSH values in this case). This suggests that by further investigating the interactions between MOF and plasmonic components, it may be possible to develop SERS substrates that surpass the performance of currently used conventional sensing platforms. The presented data for gas‐ and liquid‐phase VOC detection using MOF‐based SERS substrates provides clear evidence of the versatility and potential of these materials for practical applications.

## 4 Early Detection of Diseases through Breath Analysis

Symptomatology is a powerful approach to diseases diagnosis. However, the disease is often far advanced when symptoms manifest or become evident. At this point, valuable time has been lost, making it more difficult for the patient to overcome the disease. For that reason, the development of novel tools capable of early screening for diseases is becoming the new paradigm in clinical testing and diagnostics. In the previous section, we addressed the analytical capabilities of MOF‐based SERS substrates for VOC from both gas‐ and liquid‐phase.

**Figure 9:**
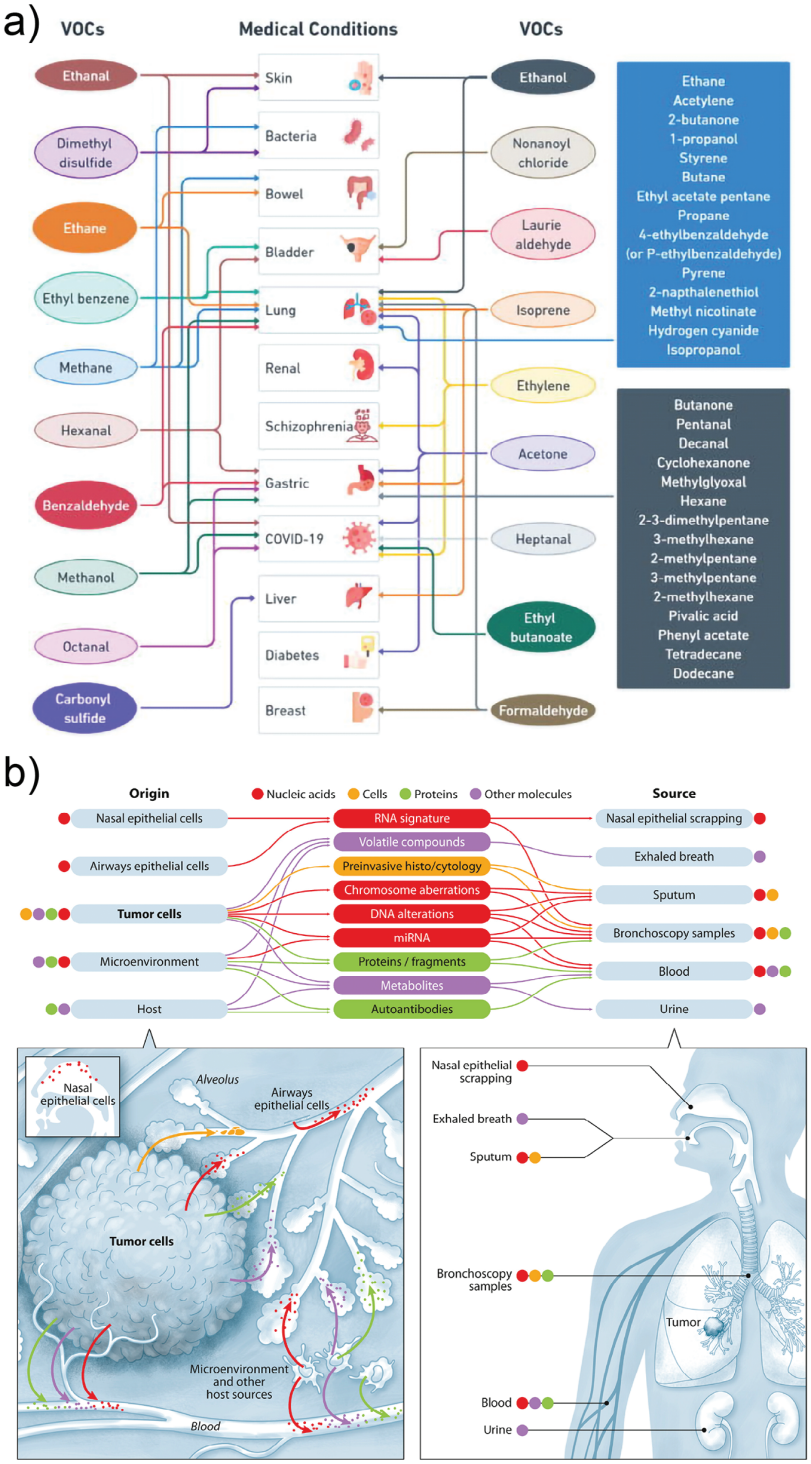
a) Map of relevant VOC associated with different medical conditions. Reproduced with permission from Zhang et al.^[^ ^123^ ^]^ Creative Commons CC BY 2023. b) Proposed biomarkers for Lung Cancer screening. Reproduced with permission from Seijo et al.^[^ ^126^ ^]^ Copyright 2019, Elsevier Inc.

Interestingly, many of these analytes are not exclusively of interest in the context of environmental and industrial monitoring but are relevant also to other application fields. For example, aldehydes and ketones are produced by our bodies through metabolic processes and can serve as indicators of our health status. In fact, there are several VOC with proved correlations to medical conditions, some of which are presented in **Figure** **9**a.^[^ ^123^ ^]^ This is particularly true for cancer diagnosis, especially for lung cancer (LC), which has one of the largest incidence and mortality worldwide.^[^ ^124^ ^]^ There are different LC diagnostic biomarkers and tools, ranging from imaging analysis to the liquid biopsy based on the analysis of autoantibodies and microRNA,^[^ ^125^ ^]^ as summarized in Figure 9b. Besides these, detection of VOC in exhaled breath represents an attractive method suitable for noninvasive diagnostics of LC and other diseases, and it has been subject of intensive research activity over the last few years.^[^ ^6^, ^7^, ^8^, ^126^, ^127^, ^128^ ^]^ Even though biomarkers associated with LC can be found in bodily fluids, VOC are generated either by or in the microenvironment surrounding the cancerous cells and being part excreted by the lungs via the exhaled breath.^[^ ^129^ ^]^ Different bioanalytical tools have been proposed for the detection of VOC for diagnostic purposes^[^ ^130^ ^]^ and among them SERS is in the spotlight due to its fingerprinting capabilities.^[^ ^55^, ^131^ ^]^ It is expected then that the combination of SERS with MOF for capturing and concentrating the VOC exhaled in breath can push forward the field.

**Figure 10:**
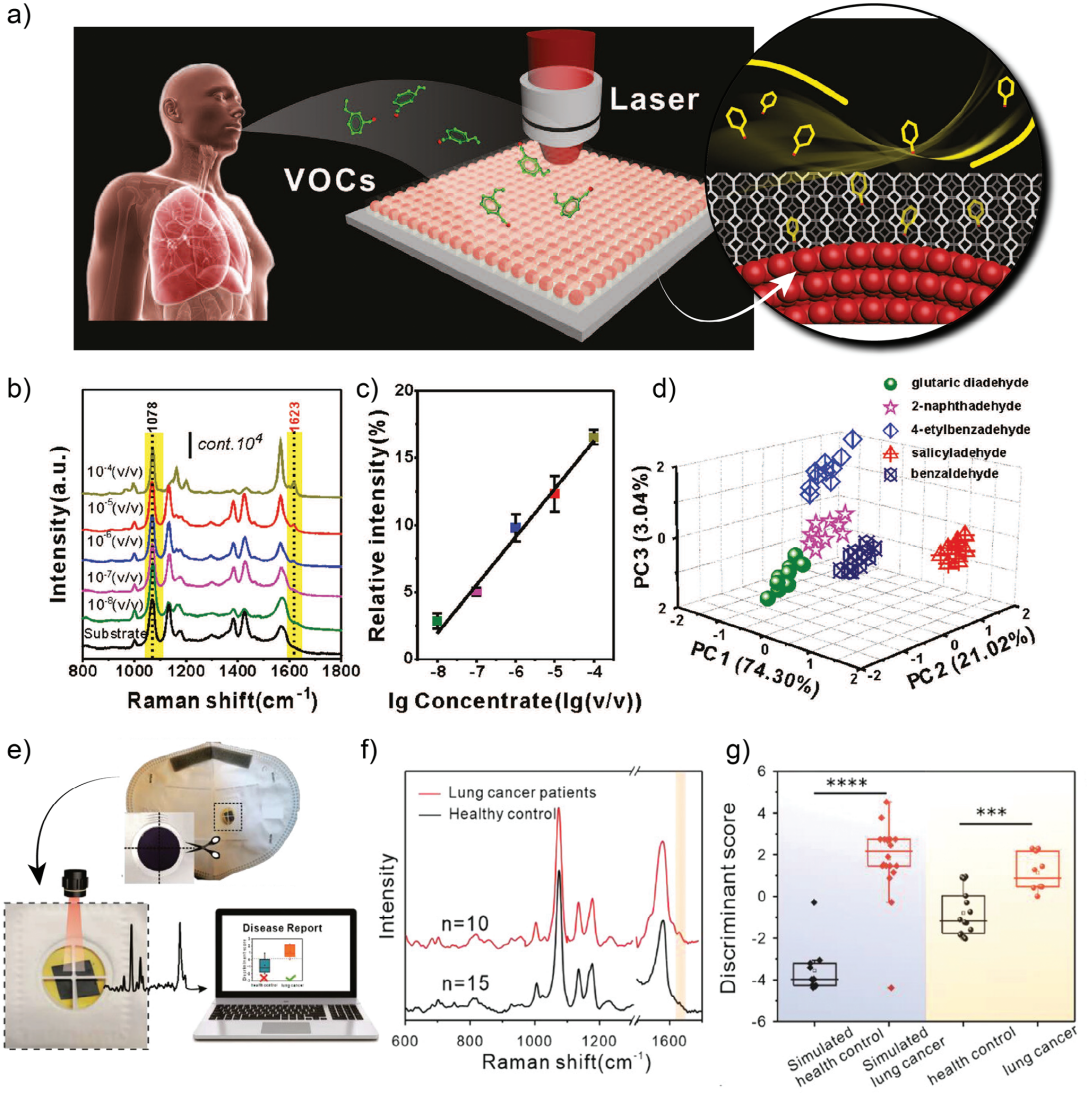
a) Schematic representation of aldehyde captures from exhaled breath for SERS detection. b) SERS spectra for 4‐ethylbenzaldehyde at different concentrations and c) the associated calibration curve. d) Principal Component Analysis (PCA) for the identification of several aldehydes. Reproduced with permission from Qiao et al.^[^ ^132^ ^]^ Copyright 2017, Wiley‐VCH Verlag GmbH & Co. KGaA. e) Picture and schematic representation of facial mask utilization for collection of VOC from breath. f) SERS spectra (averaged) for healthy and lung cancer patients. g) PC‐LDA classifier based on SERS for simulated experiments (p < 0.0001) and real samples (p < 0.001). Reproduced with permission from Li et al.^[^ ^133^ ^]^ Copyright 2022, Wiley‐VCH Verlag GmbH & Co. KGaA.

Qiao et al., synthesized gold supraparticles (GSPs, made by coalescence of individual gold NPs) and coated them with ZIF‐8 in order to yield the composite GSPs@ZIF‐8.^[^ ^132^ ^]^ In this core‐shell structure, GSPs generate plasmonic hotspots and the MOF contributes to their further spatial confinement. When employed as SERS substrate, the detection of aldehydes, one of the most well‐established biomarkers for LC, was carried out. As schematized in **Figure** **10**a, MOF‐based SERS substrate was then employed for the detection of several aldehydes including 4‐ethylbenzaldehyde, which is a classical LC biomarker. GSP were modified in this experiment by 4‐aminothiophenol, providing them with amino groups suitable for reaction with aldehydes through the so‐called Schiff reaction. The signal from the generated C═N bond upon Schiff reaction was normalized against the intensity of the Raman peak at 1078 cm^−1^ associated with C─S from the anchored thiol, serving then as internal standard (see Figure 10b,c).

The authors also discussed two important features of the SERS substrate. First, the response is not affected by humidity conditions. Although no interference from water should be expected (that is actually one of the advantages of Raman spectroscopy over, e.g., infrared spectroscopy), it could still saturate the adsorption sites on the substrate, a common issue when employing porous materials for sensing applications. However, the intrinsic hydrophobicity of ZIF‐8 micropores makes it a perfect candidate for this application. Second, the microporous ZIF‐8 layer acts also as sieving membrane and although other aldehydes could also be detected and identified (see Figure 10d), aldehydes with larger dimensions were excluded from the sensor surface probed by SERS. Even further, the SERS substrate was also proven to be highly selective toward aldehydes, not showing any signal after exposure to other small molecules that could adsorb in the ZIF‐8 porous structure, such as alcohols, organic acids, etc. Moreover, to explore the behavior of the GPS@ZIF‐8 SERS substrate and to demonstrate its suitability toward early diagnostics of LC, the substrate was exposed to simulated breath from healthy and unhealthy patients, and by Principal Component Analysis (PCA), the GSP@ZIF‐8 SERS substrate was demonstrated to be suitable for identifying the VOC signature of each simulated condition.

In a subsequent work from the same group, Li et al. demonstrate the synthesis of hollow ZIF‐8 structures encapsulating the GSPs, which improved the detection limit and reduced the interference from other molecules present during the SERS experiment.^[^ ^133^ ^]^ Also employing 4‐aminothiophenol as docking agent and internal standard, the authors transferred the SERS substrate to filter paper and integrated it into the breathing valve of a facial mask, thus generating a platform suitable for continuous collection of analytes from exhaled breath (see Figure 10e). By this approach, SERS spectra were obtained after collecting the exhaled breath of 15 healthy and 10 LC patients (Figure 10f) and analyzed by Principal Component‐linear discriminant analysis (PC‐LDA) model, trained with both simulated gas and real samples. The results, highlighted in Figure 10g, showed that the MOF‐based SERS substrate allowed for identifying LC patients with an accuracy of 60%. The use of facial masks as non‐invasive sample collection platform for point‐of‐care devices has also proven to be appropriate for the detection of SARS‐CoV‐2 infection.^[^ ^134^ ^]^ Thus developing SERS substrates integrated with Point‐of‐Care (POC) devices represents a promising and versatile platform.

**Figure 11:**
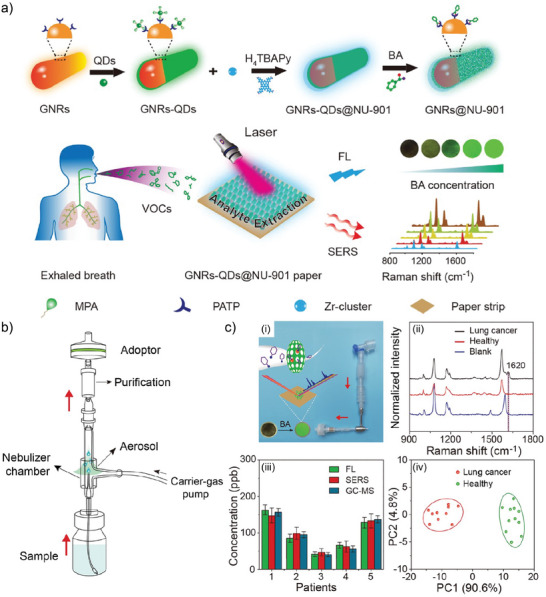
a) Schematic representation for the obtention of the GNRs‐QDs@NU‐901 dual SERS and Fluorescence substrates. MPA: 3‐mercaptopropionic acid; PATP: 4‐mercaptonoaniline; BA: Benzoic acid. b) Diagram of the nebulizer employed to convey the sample to the paper strip (dual sensor). c) ‐ (i) Photograph of breath collector and the scheme of the readout procedure after the interaction with the sample. (ii) SERS spectra for the different blank, healthy and lung cancer samples. (iii) Benchmarking of the dual fluorescence (FL) and SERS substrate against GC‐MS technique. Reproduced with permission from Xia et al.^[^ ^135^ ^]^ Copyright 2021, American Chemical Society.

An important topic, especially when considering the development of POC devices, is the availability of a facile readout of the result. For example, reactive paper strips might not provide an absolute quantification of the analyte but with a YES‐NO answer, confirming its presence with a concentration higher than certain limit. Therefore, the generation of dual sensors, capable of both identification by the naked eye and quantification are highly appealing platforms for POC devices. This was the approach followed by Xia et al., who generated Fluorescence (FL) and SERS bimodal sensor for the detection of benzaldehyde (BA).^[^ ^135^ ^]^ **Figure** **11**a provides a summary of the synthesis and readout of this FL‐SERS dual sensor. As shown, the authors started with plasmonic gold nanorods (GNRs) functionalized with 4‐mercaptonoaniline (PATP), which were later capped with CdSe/ZnS quantum dots (QDs) by self‐assembly, prior functionalization of the QDs with 3‐mercaptopropionic acid (MPA). The proximity between GNRs and QDs resulted in FRET quenching of the fluorescence of the QDs. This composite GNRs‐QDs was then included in the reaction mixture for the synthesis of NU‐901 MOF, generating single‐core‐shell GNRs‐QDs@NU‐901, later filtered through cellulose‐based paper and dried, generating the dual FL‐SERS substrates. This clever design is an example of how structure‐performance relations are of paramount importance when designing MOF‐based SERS substrates. The MOF phase acts as sieving membrane and preconcentrate the analyte (BA), favoring the Schiff reaction with PATP on the SERS‐active surface. At the same time, the addition of BA disassembles the GNRs‐QDs which lead to an increase in the fluorescence signal, thus enabling the dual detection of BA in under 5 min.

The developed GNRs‐QDs@NU‐901 SERS substrate, presented a LOD for BA of 1.2 ppb when employing the fluorescence readout, which was surpassed by the SERS readout, with a LOD of 0.1 ppb. As a proof of concept, the GNRs‐QDs@NU‐901 paper was then mounted in a holder that collected exhaled breath (Figure 11c(i)) and demonstrated the appearance of the Raman peak at 1620 cm^−1^ for LC samples (Figure 11c(ii)). After cross analyzing the samples with GC‐MS (Figure 11c(iii)) and performing PCA on the SERS readout, the authors demonstrated that the dual sensing with GNRs‐QDs@NU‐901 paper provide a suitable tool for the detection of VOC from exhaled breath without sacrificing the analytical capabilities.

There is an enormous variety of compounds in exhaled breath that can be employed for early detection of cancer, from lineal‐ and ramified‐hydrocarbons, aldehydes, ketones, and aromatic compounds with different functional groups, from simple short‐chain hydrocarbons to carboxylic acids, etc., and they can be found with different relative abundances in the range of 1–100 ppb.^[^ ^136^, ^137^, ^138^, ^139^ ^]^ As it can be seen in **Table** **3**, MOF‐based SERS substrates can perform the detection of VOC relevant for breath analysis with linear ranges and LODs well in range with the expected concentration range in real samples. In the table, the examples were classified according to SERS substrate carrying or with absent plasmonic metallic nanostructure component. This is to highlight the fact that Chemical Mechanism from MOF is indeed a valuable contribution to the overall performance of the platform since it also allows to detect VOC down to ppm levels. This can be seen for example when comparing the use of MIL‐100 (Fe) MOF for toluene detection. If no plasmonic element is present, the LOD turns to be 20 ppm; however, when included, LOD reaches a value five orders of magnitude lower (0.48 ppm).

**Table 3:** Selected examples for detection of VOC form gas phase relevant for breath analysis. All examples and references can be found in the dataset openly available in a repository.^[^ ^49^ ^]^

MOF	Metal	Analyte	Linear range [ppb]	LOD [ppb]
With plasmonic element				
ZIF‐8	Ag/Au	3‐ethyl benzaldehyde	–	1
ZIF‐8	Au	4‐ethylbenzaldehyde	0.1–1000	0.013
ZIF‐8	Au	4‐ethylbenzaldehyde	0.1–1000	7.7
ZIF‐8	Ag/Au	Benzaldehyde	–	1
NU‐901	Au‐QD	Benzoic acid	0.1–10	0.1
ZIF‐8	Au	Formaldehyde	0.1–1×10^6^	0.1
MIL‐100 (Fe)	Au	Toluene	–	0.48

Without plasmonic element				
MIL‐100 (Fe)	None	4‐ethylbenzaldehyde	–	1000
MIL‐100 (Fe)	None	Acetone	1250–3500	20 000
UiO‐66	None	Isopropanol	–	20 000
MIL‐100 (Fe)	None	Toluene	500–3500	20 000

## 5 Advanced Sensing with MOF‐Based SERS Substrates: Aromas, Flavors, and Beyond

In addition to (arguably) more established applications in industrial and environmental control and disease detection through breath analysis, MOF‐based SERS substrates are also of interest in the realm of organoleptic description. This research field deals with perceiving of aroma or taste which, in reality, is associated with a collection of molecular analytes recognized by multiple receptors in our nose and tongue, sending a complex and convoluted signal to the brain. Sometimes, the same set of analytes but in different proportions render completely different organoleptic properties. To mimic then the sense of smell and/or taste is particularly challenging because it is not ascribed to an individual analyte anymore, but a whole collection of them.^[^ ^140^, ^141^ ^]^ The detection of VOC responsible for aroma and flavor adds a unique dimension to the applicability of MOF‐based SERS substrates and it represents an exciting emerging research field.

**Figure 12:**
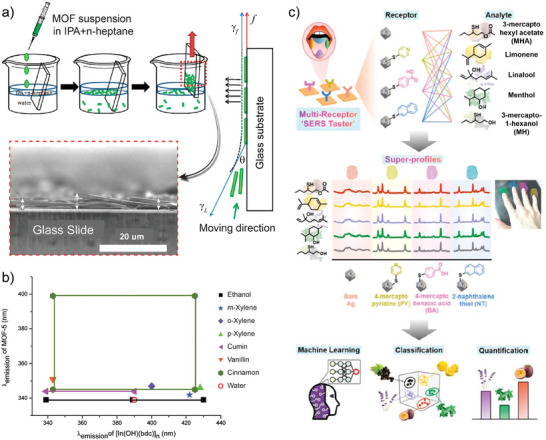
a) Schematic procedure for the synthesis of In(OH)(BDC) MOF crystals and their deposit on glass substrate by deep‐coating technique from water/n‐heptane interface, together with SEM lateral view. b) PCA readout of emission wavelength of analytes upon adsorption on In(OH)(BDC) and MOF‐5. Reproduced with permission from Lee et al.^[^ ^142^ ^]^ Copyright 2011, American Chemical Society. c) Schematic representation behind the design and operation of multireceptor SERS taster for identification and quantification of wine flavor VOC. Reproduced with permission from Leong et al.^[^ ^147^ ^]^ Copyright 2021, American Chemical Society.

The use of MOF‐based sensors for the detection of odorants (i.e., VOC responsible for an aroma perception) was first reported by Lee et al.^[^ ^142^ ^]^ Although their study does not employ SERS as the readout technique, it is of relevance to include it in this section to discuss the important aspects regarding VOC detection for aroma profiling. In their work, the authors have synthesized microcrystals of indium‐based luminescent MOF In(OH)(BDC), which were then confined in a water/n‐heptane interface (see **Figure** **12**a). Then, a glass substrate was placed vertically in the mixture, and as the n‐heptane evaporated, the MOF microcrystals self‐assembled on the glass substrate. This process produced a film with >80% coverage of the glass slide, with thickness between 3–5 µm (depending on the overlapping of the microcrystals). The advantage of working with In(OH)(BDC) MOF is that upon excitation (in this case, with a 270 nm wavelength light source), the crystals exhibited a photoluminescent (PL) peak at 326 nm, probably rising from ligand‐to‐metal charge transfer process. Upon adsorption of the odorant, the π system associated with the charge transfer is disrupted, thus redshifting the PL peak, which was then used as transducing mechanism for the adsorption process. Now, as stated previously, when aiming to describe a complete aroma, there is a need for detecting more than just one adsorption event, but rather a specific pattern. Therefore, the authors employed also MOF‐5 crystals which exhibited a blue shift upon adsorption of the target analytes. Having then a set of two MOF responding differently to the same substances, the PL peak shift for each of them was used as component in a PCA analysis, mapping the response toward the analytes, and generating a pattern recognition for the aroma, as it can be seen in Figure 12b. This strategy not only allowed for the detection of the particular odor of, e.g., cumin or cinnamon, but also enabled the discrimination between structural isomers such as m‐xylene, o‐xylene, and p‐xylene. For proving the versatility of this system, the photoluminescent In(OH)(BDC) MOF was also employed in a later work toward the development of a “biomimetic tongue”, thus aiming to mimic the sense of taste.^[^ ^143^ ^]^ This MOF was also modified with (+)‐polyaniline, mimicking the activity of guanine nucleotide‐binding protein receptor involved in taste transduction.^[^ ^144^ ^]^

In a related approach, SERS was employed in several works toward the detection of odorants.^[^ ^145^, ^146^ ^]^ Fairly recently, this method was explored in the development of a “biomimetic tongue” as a complement to a “biomimetic nose”. Contrary to the previous example (where MOF was employed without using SERS as readout), the work of Leong et al. rely in SERS alone for the development of a multiplexing platform for identifying wine flavors.^[^ ^147^ ^]^ The strategy followed by the authors is schematized in Figure 12c, where it can be seen that different chemical modifications of Ag nanocubes assembled by Langmuir–Blodgett technique (acting as receptors) allowed generation of the so‐called SERS super‐profiles. Then, by employing machine‐learning‐driven chemometric models, the authors demonstrate not only the identification but also quantification of wine flavors.

More into the details, Leong et al. chose five target analytes, as archetypical descriptors of typical wine flavors: menthol for aliphatic alcohols, linalool and limonene for terpenes, and 3‐mercaptohexyl acetate and 3‐mercapto‐1‐hexanol for sulfur‐bearing compounds. Then, the spectral variations on the SERS response associated with the differential interaction of the functional groups of the analytes and the functional groups introduced on the Ag nanocubes surface, were employed for PCA and Support Vector Machine Discriminant analysis (SVM‐DA) analysis. The results showed that the SERS substrate was able to classify all the flavors with 100% accuracy. Finally, the SERS substrates were employed for the multiplex analysis of artificial wine matrices, prepared with water, ethanol, glycerol, and tartaric acid. Then, the authors analyzed fourteen combinations of different flavor concentrations and employed the associated SERS super‐profiles as calibration and validation sets, and later tested with six “unknown” samples, which were quantified with 95–100% accuracy.

Through this discussion, we have shown that MOF and SERS, individually, hold a potential toward the development of sensing platforms for aromas and flavors profiling. It is logical to assume that the combination of both approaches, i.e., the generation of MOF‐based SERS substrates will provide a powerful approach to this field. To the best of our knowledge, there is only one report addressing the use of MOF‐based SERS substrates toward such a goal.

**Figure 13:**
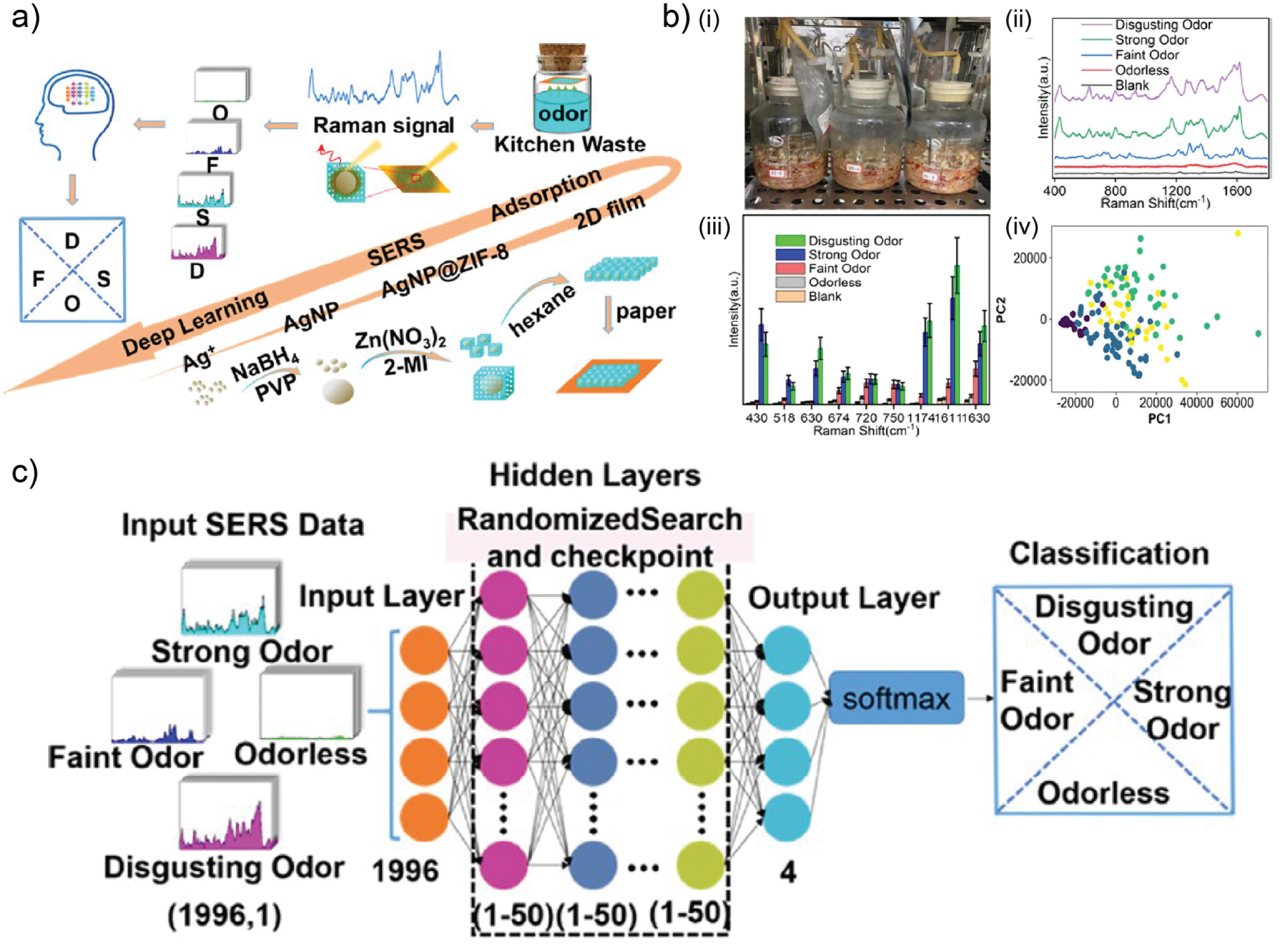
a) Schematic representation of the odor intensity from kitchen waste autograding process, starting with the MOF‐based SERS substrate synthesis. b) ‐ (i) Picture of the odor collection approach, together with (ii) SERS spectra, (iii) peak intensities comparison, and (iv) PCA mapping for the different odor intensities. c) Schematic representation of the training of DL model employed for odor classification. Reproduced with permission from Yu et al.^[^ ^148^ ^]^ Copyright 2022, American Chemical Society.

In their work, Yu et al. explored the generation of AgNP@ZIF‐8 later integrated in a paper substrate for detecting the odor intensity generated by kitchen waste.^[^ ^148^ ^]^ The authors combined Ag nanoparticles encapsulated in ZIF‐8 shells with controllable thicknesses (from 20 to 100 nm). As schematized in **Figure** **13**a, the SERS‐active core‐shell particles were transferred to a filter paper from a nanofilm generated in an organic solvent – water interface. This MOF‐based SERS substrate was then exposed to odor sources and analyzed by SERS in conjunction with deep‐learning (DL) model to classify the odor intensity into four categories: Odorless, Faint, Strong, and Disgusting odor. Specifically, kitchen waste odor was simulated by collecting different typical residues, from rice, eggshells, meat, vegetables, paper and plastic, and placed into a container with a rubber plug (see Figure 13b(i)). The MOF‐based SERS substrates were then exposed to the gas generated from this mixture and the SERS spectra were recorded after different exposure times (Figure 13b(ii)). By recording the Raman intensity of different peaks for each sample and performing a PCA (Figure 13b(iii),(iv)), the authors showed that the system presented a good autograding capability toward the distinction of each of the odor intensity levels. The SERS data from known samples was then employed to train the DL model (Figure 13c); using 96 SERS spectral data as training set, 25 SERS spectral data as verification set, and 31 SERS spectral data as test set, DL approach succeeded on the identification of smell intensity with a >99% accuracy. This is a promising result that highlights the potential of MOF‐based SERS substrates for VOC detection in different fields, which can be translated to solving daily‐life‐problems.

## 6 Conclusion and Perspectives

Over the past decade, research fields of MOF and SERS rapidly progressed and only recently they merged into new analytical platforms that are particularly well suited for the analysis of VOC. Throughout this review, the intricate interplay of both MOF and metallic nanostructures is discussed, together with important design rules enabling maximizing performance of MOF‐based SERS substrates. This includes the way in which MOF grow on a metallic surface that is dictated not only by the nature of the MOF itself, but also by the metallic nanostructure that impacts on the internal structure of the MOF phase, subsequently affecting the permeation of analytes toward the SERS‐active surface. Therefore, understanding of this complex interplay is essential for successful design, characterizing, and optimizing the performance of MOF‐based SERS substrates to ensure a strong amplification of optical signal and allow to efficiently preconcentrating the target analyte species within the plasmonic hotspots.

Our review covers the use of substrates that combine MOF and metallic nanostructures for SERS – based detection of VOC in three main contexts: environmental and industrial monitoring, early disease detection through breath analysis, and emerging field of aromas and flavors profiling. Such a diversity of application areas underscores the versatility, robustness, and potential of serving in future commercially viable analytical technologies. However, several challenges remain to be solved to harness the attractive features of MOF‐based SERS substrates and capitalize on the already performed research. In particular, the preparation means suitable for scaling up needs to be established without compromising the well‐defined and reproducible plasmonic and chemical features endowed to the substrates. Moreover, developing tools for a systematic selection of the appropriate MOF‐substrate pair are needed, considering the particular properties of target analytes, suitable amplification mechanism, and adsorption capacity of the porous framework, not only in terms of surface area but also pore accessibility and pore chemistry. Depending on the application, the platform should be able to analyze patterns in concentration changes of analytes and provide ‐ideally‐ with a result at two different levels: identification and quantification of the target analyte(s). Regardless of the field of application, different aspects must be kept in mind and require further research efforts to push further the field of VOC detection by MOF‐based SERS platforms into real‐life scenarios: i) Stability: given their coordinative nature, some MOF are susceptible to harsh environments, which might limit their on‐site applicability. Similarly, plasmonic properties of the SERS substrate might also be affected. ii) Sensitivity: despite their large surface areas and tunable porosity, specific analyte‐MOF‐substrate combinations are needed to maximize the sensitivity of the platform, especially when aiming for quantification of trace amounts of analytes. iii) Selectivity: fingerprinting capabilities of SERS ensure that highly specific interaction between the substrate and the analyte is not needed for its detection. However, SERS signal is affected by (among other factors) location, orientation, and surface coverage of the analyte on the SERS‐active sites. Therefore, strategies that minimizes blocking of the sensor surface are needed. This is (partially) solved using MOF, since their regular and well‐defined microporosity serves as a molecular sieve. Nevertheless, discrimination between VOC with similar chemical structures can be challenging, depending on the complexity of the sample matrix. iv) Reproducibility: MOF synthesis and SERS‐active substrate fabrication have intrinsic variability that must be accounted for in order to provide a uniform and regular response. Despite that, the current state‐of‐the‐art offers significant robustness in this regard. For instance, our review of the literature reveals a mean Relative Standard Deviation (RSD, %) of 10%, indicating the high reproducibility of MOF‐based SERS substrates. It is worth noting though that only 35% of the entries in our dataset include an RSD value. This led to the final aspect highlighted here, which is (v) the need for establishing robust, consistent, and thorough methodologies and standardized report guidelines, pushing forward the advancement of the field.

The current performance of MOF‐based SERS platforms holds potential for the implementation of a new generation of sensors capable to perform on‐site, continuous monitoring analysis indoor and in the workspace, thus ensuring a safe environment; for developing continuous environmental monitoring platforms; and to advance diseases diagnostics and find a place along the different diagnostic tools in clinical settings. Finally, MOF‐based SERS platforms are promising candidates in the context of mimicking the sense of smell and taste, enabling complex mixture profiling.

Despite the challenges, MOF‐based SERS substrates have proven to be a versatile, robust, and powerful tool, benefiting from the unique properties of MOFs with the exceptional sensing performance of SERS. The interdisciplinary nature of this research, merging chemistry, materials science, nanotechnology, and plasmonic, with other fields like diagnostics, is a thrilling aspect of this field, pushing the limits of not only VOC detection but also other analytes present in trace amounts in complex and variate matrices.

## Conflict of Interest

The authors declare no conflict of interest.
